# HIV-1 latency reversal and immune enhancing activity of IL-15 is not influenced by sex hormones

**DOI:** 10.1172/jci.insight.180609

**Published:** 2024-09-10

**Authors:** Carissa S. Holmberg, Callie Levinger, Marie Abongwa, Cristina Ceriani, Nancie M. Archin, Marc Siegel, Mimi Ghosh, Alberto Bosque

**Affiliations:** 1 Department of Microbiology, Immunology, and Tropical Medicine, George Washington University, Washington DC, USA.; 2 UNC HIV Cure Center and; 3 Department of Medicine, The University of North Carolina at Chapel Hill, Chapel Hill, North Carolina, USA.; 4 The George Washington School of Medicine and Health Sciences, Washington DC, USA.; 5 Department of Epidemiology, George Washington University, Washington DC, USA.

**Keywords:** AIDS/HIV, Immunology, Cytokines, Sex hormones

## Abstract

The role of different biological variables including biological sex, age, and sex hormones in Human immunodeficiency virus (HIV) cure approaches is not well understood. The γc-cytokine IL-15 is a clinically relevant cytokine that promotes immune activation and mediates HIV reactivation from latency. In this work, we examined the interplay that biological sex, age, and sex hormones 17β-estradiol, progesterone, and testosterone may have on the biological activity of IL-15. We found that IL-15–mediated CD4^+^ T cell activation was higher in female donors than in male donors. This difference was abrogated at high 17β-estradiol concentration. Additionally, there was a positive correlation between age and both IL-15–mediated CD8^+^ T cell activation and IFN-γ production. In a primary cell model of latency, biological sex, age, or sex hormones did not influence the ability of IL-15 to reactivate latent HIV. Finally, 17β-estradiol did not consistently affect reactivation of translation-competent reservoirs in CD4^+^ T cells from people living with HIV who are antiretroviral therapy (ART) suppressed. Our study has found that biological sex and age, but not sex hormones, may influence some of the biological activities of IL-15. Understanding how different biological variables may affect HIV cure therapies will help us evaluate current and future clinical trials aimed toward HIV cure in diverse populations.

## Abstract 2

In this work we evaluated whether biological sex, age, and sex hormones influenced the activity of the cytokine IL-15 used in HIV cure approaches

## Graphical abstract

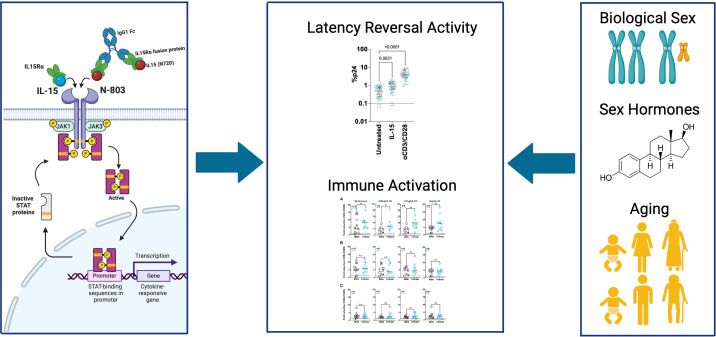

## Introduction

Human immunodeficiency virus (HIV) has caused over 40 million deaths in the last 4 decades with an estimated 38 million people living with HIV (PWH), with women, and younger women particularly, disproportionately affected (1, 2). While antiretroviral therapy (ART) prevents progression of HIV into acquired immunodeficiency syndrome (AIDS), ART is not curative, as HIV persists in latent reservoirs (3–5). Further, PWH experience chronic immune activation and inflammation with a higher incidence of non-AIDS–related comorbidities, which again disproportionately affect women living with HIV (6, 7).

To achieve a cure, a strategy to target the latent reservoir termed “shock and kill” utilizes a Latency Reversing Agent (LRA) to activate transcription of latent HIV with the consequent killing of the reactivated cells by either the immune system or direct cytopathic effects from HIV. Several LRAs have reached clinical trials, including histone deacetylase inhibitors, toll-like receptor agonists, and the γc-cytokine IL-15 superagonist N-803 (8–11). LRAs that also promote immune clearance of the latent reservoir are an important component toward ensuring eradication of HIV infection (12, 13). IL-15 has demonstrated both latency reversal and immune enhancing activity by showing reactivation of latent HIV both ex vivo and in vivo, improved HIV-specific CD8^+^ T cell responses and cytotoxic function, and enhanced HIV-specific NK cell effector function (14–21). Recently, a phase I clinical trial showed safety of the IL-15 superagonist N-803 in PWH (22). There was variability in N-803 activation of CD8^+^ T and NK cell responses and in the reduction of the inducible HIV reservoir among the participants; however, this trial had only 16 participants, all of whom were male (22).

Sex hormones have several functions in the body including effects on the immune system, which may impact infectious diseases including HIV (23, 24). Previous studies have found influences of 17β-estradiol (E2) and estrogen receptor-1 (ESR-1) signaling on the inducible HIV reservoir (25, 26). Additionally, differences in HIV-1 acquisition and disease progression have been shown between men and women (27, 28). Further, cisgender females have a lower inducible HIV-1 RNA than cisgender males (25, 26, 29). However, no differences have been seen in HIV-1 DNA between sexes (26, 29, 30). In addition to the potential impact of biological sex and sex hormones on HIV cure strategies, with the ability of HIV to be successfully suppressed with ART, the average age of PWH is starting to increase. A recent study has shown differences in the inducible reservoir with biological sex and age, with an observed increase in inducible reservoir in women after menopause (29). Overall, the potential biological impact of sex differences, sex hormones, and age are important factors to consider when designing HIV cure strategies and clinical trials. This study aims to determine the effects of biological sex, age, and sex hormones on the activity of IL-15 as an immune activator and LRA.

## Results

### Effects of sex hormones, biological sex, and age on IL-15–mediated immune activation.

**Figure 1:**
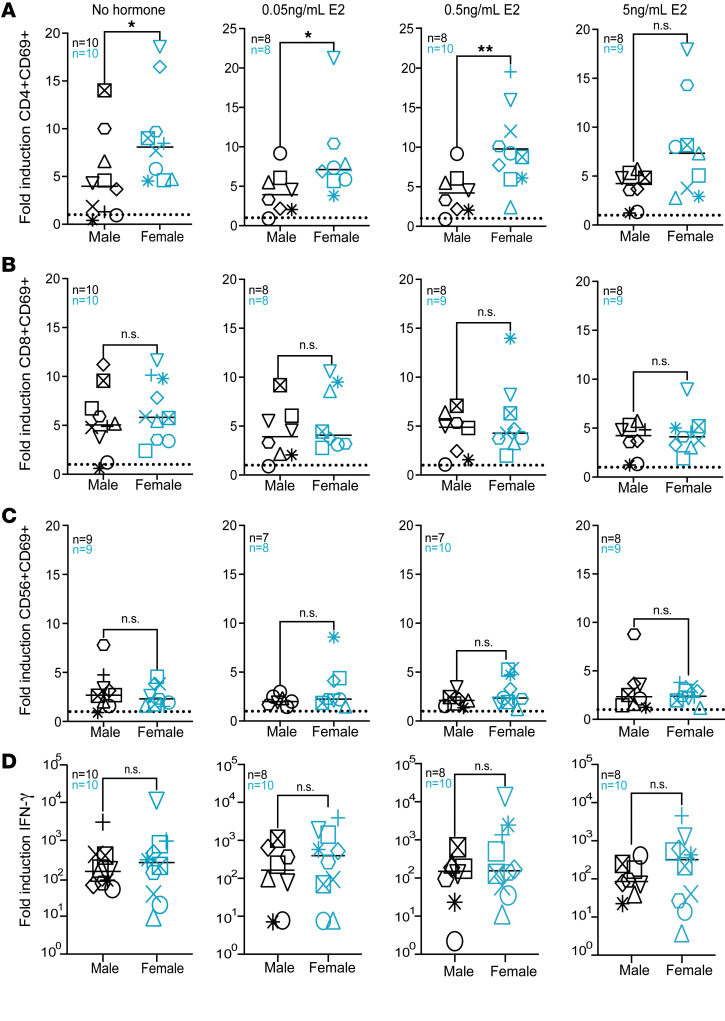
Biological sex influences IL-15–mediated immune activation of CD4^+^ T cells. . Comparative analysis of immune activation mediated by IL-15 (100 ng/mL) in 10 age-matched male and female participants (A) CD4^+^ T cells, (B) CD8^+^ T cells, (C) NK cells, and (D) IFN-γ production with increasing concentrations of estradiol (E2), as indicated. Teal symbols are female donors and black symbols are male donors; each individual participant is designated by their own shape in each graph. Mann-Whitney test was used to calculate *P* values (*P < 0.05). ROUT outlier test was used to remove outliers

IL-15 induces immune activation of lymphocytes and has been shown to enhance NK and CD8^+^ T cell activity against HIV (15, 19, 31). To determine the influence of sex hormones on IL-15–mediated immune activation, we treated peripheral blood mononuclear cells (PBMCs) from HIV seronegative donors overnight with 17β-estradiol (E2), progesterone (P4), or the testosterone derivative danazol, and then stimulated with IL-15 (100ng/mL) for 48 hours. Previous studies have shown the presence of estrogenic compounds in media with phenol red and FBS (32, 33). To remove any influence from these estrogenic compounds, RPMI media without phenol red and charcoal-stripped serum was used (referred to as CSS media). We determined hormone concentrations based on physiological serum levels found in cisgender males and females over their lifespan. For E2, we used concentrations at 0.05 ng/mL, 0.5 ng/mL, and 5 ng/mL to cover the range found in plasma in males (between 10 and 82 pg/mL), accounting for menstrual cycle shifts (up to 0.2 ng/mL during follicular development) and pregnancy (up to 20 ng/mL during the third trimester) (34–37). We used 0.25 ng/mL, 2.5 ng/mL, and 25 ng/mL to cover P4 levels in males (< 0.20 ng/mL) (38); during menopause (< 0.4ng/mL); and during the luteal phase of the menstrual cycle (up to 25 ng/mL) (34). Finally, we used the testosterone derivative danazol at 0.3 ng/mL, 3 ng/mL, and 30 ng/mL to cover the levels of bioavailable testosterone found in younger male adults (3 ng/mL), older males (1.5 ng/mL), and females (0.2 mg/mL) (39–41). We performed this assay in 10 male and 10 female age-matched donors (Supplemental Table 1; supplemental material available online with this article; https://doi.org/10.1172/jci.insight.180609DS1). Immune activation was analyzed in CD4^+^ T, CD8^+^ T, and NK cells by flow cytometry. While we observed varying levels of activation among donors, treatment with these hormones at any concentration did not impact levels of IL-15–mediated activation of CD4^+^ T, CD8^+^ T, or NK cells in either male or female donors (Supplemental Figures 1 and 2). We further analyzed for differences in activation by biological sex. IL-15 activation of CD4^+^ T cells showed higher activity in female donors than in male donors (Figure 1A). Interestingly, this sex difference was not observed in CD8^+^ T cells or NK cells (Figure 1, B and C). This difference in IL-15–mediated activation of CD4^+^ T cells was abrogated at higher levels of E2 (5 ng/mL), but the effect of P4 and danazol varied among the different concentrations tested (Figure 1A and Supplemental Figure 3).

Since we observed that IL-15 induced higher immune activation of CD4^+^ T cells from female donors than from male donors, we hypothesized that differences may exist in the IL-15 signaling pathway. IL-15 signals through the JAK/STAT pathway phosphorylating STAT5, among others (42–45). To investigate the potential mechanism behind higher CD4^+^ T cell activation in female donors, we analyzed STAT5 phosphorylation (pSTAT5) by Western blot in total CD4^+^ T cells after overnight stimulation with IL-15 (100 ng/mL) in CSS media. We observed STAT5 activation (measured as pSTAT5) in both female donors and male donors, however, no significant difference was observed (Supplemental Figure 4).

Besides immune activation, IL-15 can promote the induction of several cytokines, including TNF-α and IFN- γ (42, 44). We therefore evaluated the potential impact of sex hormones, biological sex, and age on IL-15–mediated cytokine production. We analyzed IL-15–mediated cytokine production after 48 hours stimulation with a 10-cytokine panel array (IFN-γ, IL-1β, IL-4, IL-5, IL-6, IL-8, IL-10, IL-12p70, IL-22, and TNF-α). IL-15 induced the production of all 10 cytokines (Supplemental Figure 5). Sex hormone treatments with E2, P4, or danazol showed no difference in IL-15–mediated induction for all 10 cytokines compared with no hormone treatment (Panels A and B of Supplemental Figures 6–15). As with CD8^+^ T and NK cell activation, biological sex did not influence IFN-γ or any of the other 9 cytokines at any of the E2 concentrations tested (Figure 1D and Panel C of Supplemental Figures 7–15).

We further assessed the potential impact of sex hormones on other IL-15 activities, including CD4^+^ T cell proliferation and granzyme B production, which are important for anti-HIV activity (20). First, the same supernatants that were used in the cytokine analysis were further analyzed for granzyme B production. We did not observe any changes in granzyme B production with any of the sex hormones in male or female donors (Supplemental Figure 16). Next, we measured the effects of E2, P4, or danazol on IL-15–mediated proliferation in CSS media of memory (CD45R0+) and naive (CD45R0–) CD4^+^ T cells using CellTrace Yellow for 7 days. We observed no changes in the rates of IL-15–induced proliferation by any of the sex hormone treatments used (Supplemental Figure 17).

**Figure 2:**
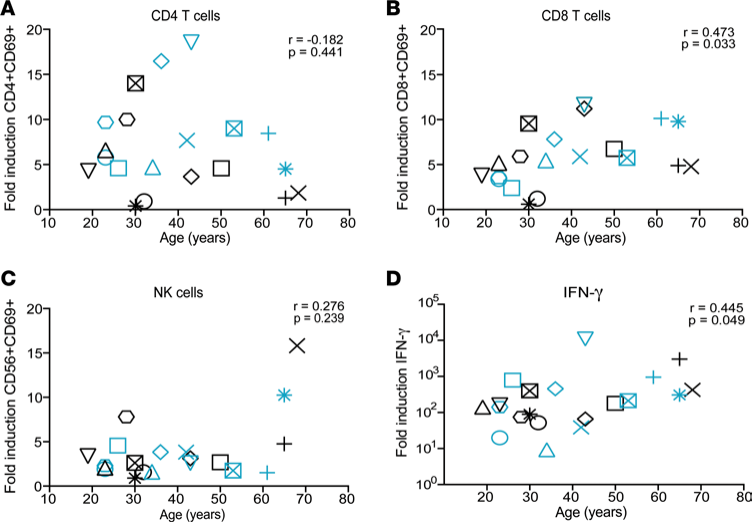
Age influences IL-15–mediated CD8^+^ T cell activation and IFN-γ production. Fold induction of immune activation versus donor age for (**A**) CD4^+^ T cells, (**B**) CD8+ T cells, (**C**) NK cells, and (**D**) IFN-γ production. Teal symbols are female donors and black symbols are male donors; each individual participant is designated by their own shape in each graph. Nonparametric spearman correlation was used to calculate r and *P* values. *n* = 20 for all graphs.

Finally, aging has been associated with a decline in immune response (46–48). However, little is known regarding the influence of age on IL-15 activity. Interestingly*,* IL-15–mediated activation of CD8^+^ T cells was positively correlated with age (Figure 2B). No correlation with age was observed in either CD4^+^ T or NK cell activation (Figure 2, A and C). Additionally, there was a positive correlation between the production of IFN-γ and age but not for any of the other 9 cytokines (Figure 2D, Panel C of Supplemental Figures 7–15).

### Expression of estrogen, progesterone, and androgen receptor in immune cells.

**Figure 3:**
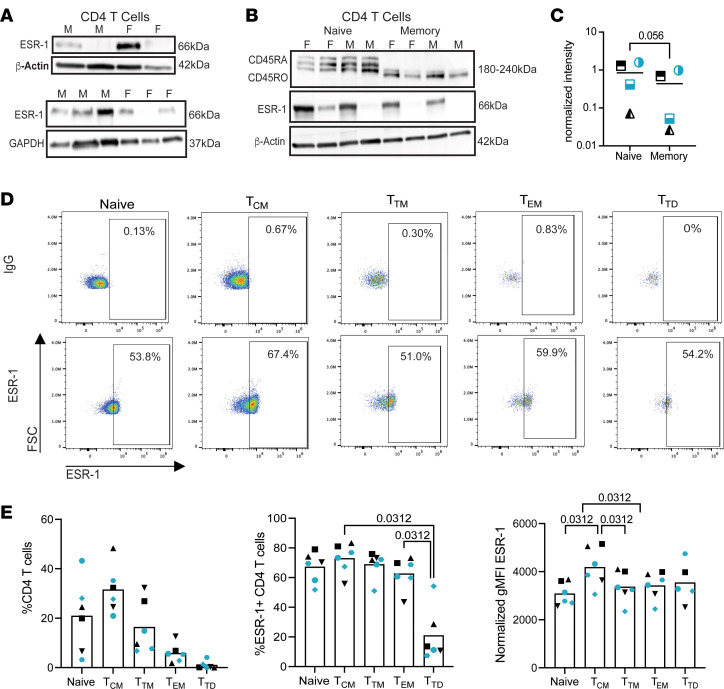
Sex hormone receptor expression in CD4^+^ T and CD8^+^ T cells. (**A**) Western blot analysis of estrogen receptor-1 (ESR-1, clone EPR4097, Abcam, ab108398) in 2 male and 2 female donors in total CD4^+^ T cells (top panel), and in additional 3 male and 3 female donors (bottom panel) (*n* = 10). (**B**) ESR-1 analysis in CD4^+^ T cell naive and memory subsets in 2 male and 2 female donors. Purity of the populations evaluated using CD45 isoforms with naive CD4^+^ T cells (CD45RA) showing longer isoforms than memory (CD45RO) cells. (**C**) Relative quantification of ESR-1 in naive and memory CD4^+^ T cell purified populations. Mann-Whitney test was used to calculate *P* value (*n* = 4). (**D**) Representative gating of ESR-1 expression in CD4^+^ T cell subsets with the top row showing IgG control antibody and the bottom showing ESR-1 antibody. (**E**) Quantification of the percentage of each subset with the total CD4 population (left). Analysis of the percent ESR-1 expression in each CD4 subset (middle) and the normalized MFI of ESR-1 in each subset (right). *n* = 6. Wilcoxon matched-pairs signed rank test was used to calculate *P* values. Teal symbols are female donors and black symbols are male donors; each individual participant is designated by their own shape in each graph.

It has been shown that ESR-1 is expressed in CD4^+^ T cells by RNA, measured by RT-PCR and IHC (49, 50). However, ESR-1 protein expression in CD4^+^ T cells has not been consistently observed. We measured protein expression of sex hormone receptors ESR-1, progesterone receptor (PR), and androgen receptor (AR) in CD4^+^ T, CD8^+^ T, and NK cells by Western blot. We observed low expression of ESR-1 in CD4^+^ T, CD8^+^ T, and NK cells when compared with the ESR+ breast cancer cell line T47D (Supplemental Figure 18). Additional analysis using a more sensitive ESR-1 antibody showed evidence of ESR-1 expression in total CD4^+^ T cells but was variable by donor (Figure 3A). Because of the differences in ESR-1 expression among donors in total CD4^+^ T cells, we next wanted to determine if there was a difference in ESR-1 expression between naive and memory CD4^+^ T cell subsets in male and female donors. We observed expression of ESR-1 in both naive and memory CD4^+^ T cell subsets with variable expression in both male and female donors (Figure 3, B and C). Due to the low detection of ESR-1 detected by Western blot, we developed a flow cytometry assay using SP1 clone against ESR-1 conjugated to fluorochrome Alexa Fluor647. We first validated this antibody using the ESR+ cell line T47D (Supplemental Figure 19). Then, we evaluated ESR-1 expression in naive, central memory (T_CM_), transitional memory (T_TM_), effector memory (T_EM_), and terminally differentiated memory (T_TD_) CD4^+^ T cells (Figure 3D and Supplemental Figure 19). We observed a similar percentage of cells expressing ESR-1, except T_TD_, which had lower expression; moreover, this subset was the minority subset (Figure 3E, left and middle). Interestingly, T_CM_ expressed the highest levels of ESR-1 expression relative to the other populations (Figure 3E, right). We next evaluated whether IL-15 can change the expression of ESR-1 in CD4^+^ T cells. We observed an overall reduction of ESR-1 expression in all subsets after 24 hours in culture, but IL-15 did not influence the expression of ESR-1 (Supplemental Figure 20). Regarding PR, expression was detected in all 3 cell types (Supplemental figure 18). PR has distinct functional isoforms PR-A, PR-B, and PR-C, generated through alternative splicing, that have different biological functions (51, 52). PR-C, an N-terminus truncated isoform, may have a regulatory function shown in vitro and a function during pregnancy (52). CD4^+^ T cells showed expression of isoform C in all 4 donors (Supplemental Figure 18B, top panel) (52, 53). CD8^+^ T cells showed low expression of isoform A and expression of isoform C in 2 donors (Supplemental Figure 18B, middle panel). Lastly, NK cells showed varying expression of all isoforms A, B, and C in the 4 donors (Supplemental Figure 18B, lower panel). Finally, AR was expressed in CD4^+^ T and CD8^+^ T cells, but low expression was observed in NK cells (Supplemental Figure 18C).

### Sex hormones do not influence reactivation of latent HIV in the T_CM_ model of latency.

**Figure 4:**
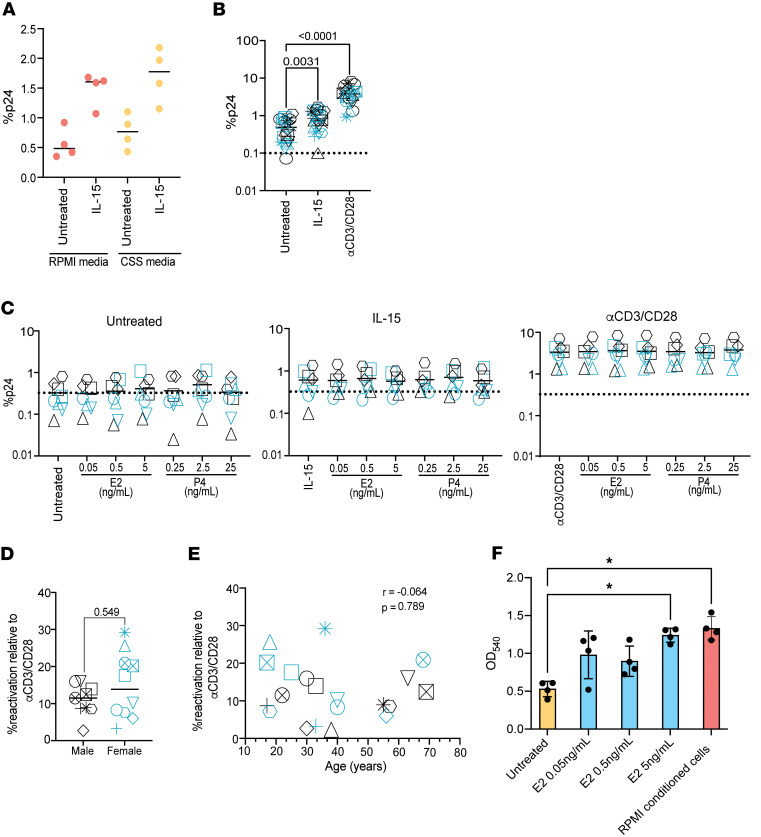
Biological sex, age, and sex hormones do not influence IL-15–mediated latency reversal in a primary CD4^+^ T cell model of HIV latency. (**A**) Reactivation analysis using the T_CM_ model of latency comparing IL-15–mediated reactivation in RPMI media versus CSS media. *n* = 4. (**B**) Reactivation analysis using the T_CM_ model of latency with 100 ng/mL IL-15 or αCD3/CD28 of 10 male and 10 female donors (*n* = 20). Friedman multiple comparison test was used to calculate *P* values. (**C**) Reactivation analysis using the T_CM_ model of latency of 4 male and 4 female donors treated with 3 concentrations of E2 and P4 in unstimulated (left), IL-15 stimulated (middle), and αCD3/CD28 stimulated (right) conditions. Percent reactivation of IL-15 relative to αCD3/CD28 comparing male versus female donors (**D**) and correlation with age (**E**) (*n* = 20). Mann-Whitney test and nonparametric spearman correlation were used to calculate *P* values. (**F**) Proliferation of T47D in CSS media supplemented with increasing concentration of E2 and compared with positive control RPMI media (*n* = 4). Kruskal-Wallis test was used to calculate *P* values (*<0.5). Teal symbols are female donors and black symbols are male donors; each individual participant is designated by their own shape in each graph.

Previous studies showed reactivation of latent HIV was affected by 17β-estradiol in a Jurkat and primary cell model of latency (25, 26). We tested the effects of 17β-estradiol and progesterone on IL-15– and αCD3/CD28–mediated HIV reactivation in the T_CM_ model of latency (54, 55). For this study, we used CSS media during reactivation to avoid estrogenic compounds found in serum and phenol red (32, 33). First, we confirmed that CSS media does not affect viral reactivation compared with RPMI media mediated by IL-15 in this model (Figure 4A and Supplemental Figure 21). Next, latently infected cells were pretreated (in the presence of ART) with E2 (0.05 ng/mL, 0.5 ng/mL, and 5 ng/mL) or P4 (0.25 ng/mL, 2.5 ng/mL, and 25 ng/mL) for 2 hours in CSS media and then treated with IL-15 (100 ng/mL) or positive control αCD3/CD28. Reactivation was assessed by flow cytometry on day 19 by quantitating cells expressing p24 and downregulating CD4. We generated latently infected cells from 10 male and 10 female age-matched donors (Supplemental Table 2). Both IL-15 and αCD3/CD28 induced HIV reactivation in the presence of CSS media (Figure 4B). However, we did not observe any influence of E2 or P4 on HIV reactivation by either IL-15 or αCD3/CD28 stimulation (Figure 4C). Further, viral reactivation by IL-15 was not influenced by either biological sex (Figure 4D) or age (Figure 4E) in this model. Since we did not observe any effects of E2 on latency reversal, differing from results reported by others (25), we wanted to confirm the bioactivity of the E2 used. For that, we performed a growth assay using the breast cancer cell line T47D, which requires E2 for proliferation. E2 treatment increased growth of T47D in a dose-dependent manner, indicating that the E2 used in our study was biologically active (Figure 4F).

### 17β-estradiol does not consistently influence reactivation in PWH who are ART-suppressed.

**Figure 5:**
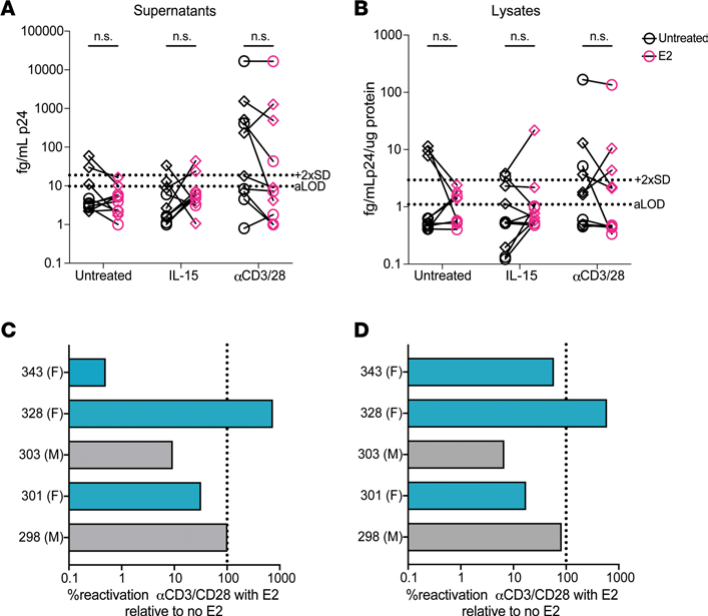
Varying effects of 17β-estradiol on HIV reactivation in PWH who are ART suppressed. (**A** and **B**) Viral reactivation was measured in cells isolated from 5 male and 5 female ART-suppressed PWH using an ultrasensitive p24 ELISA in (**A**) supernatants or (**B**) cell lysates of total CD4^+^ T cells treated with 100 ng/mL IL-15 or αCD3/CD28 with or without 300 pg/mL of E2. Pink symbols are with E2 treatment and black symbols are no E2 treatment. Diamonds are female participants and circles are male participants. Multiple Wilcoxon tests were used to calculate *P* values. (**C** and **D**) Percentage reactivation of αCD3/CD28 treated with E2 relative to αCD3/CD28 without E2 in (**C**) supernatants and in (**D**) cell lysates in the 5 participants that responded to αCD3/CD28. Teal bars are female participants and grey bars are male participants.

Based on the lack of effect of E2 in the primary cell model of latency, we next wanted to test whether E2 affects viral reactivation of translation-competent latent reservoirs from PWH who are ART-suppressed. Total CD4^+^ T cells were isolated from 5 male and 5 female participants (Supplemental Table 3) and pretreated with 300 pg/mL E2 for 2 hours in the presence of ART and in CSS media before adding 100 ng/mL IL-15 or αCD3/CD28. Supernatants and cell lysates were collected 96 hours later and analyzed for p24 by ultrasensitive ELISA (56). In both supernatants and lysates, we observed low levels of reactivation with IL-15 treatment and varying response with αCD3/CD28 treatment (Figure 5, A and B). There were 5 participants who were responders to αCD3/CD28-mediated reactivation, showing measured p24 above the limit of detection of the assay (Figure 5, A and B). Among participants who responded to αCD3/CD28 stimulation, there were no clear differences in response by biological sex, albeit there were not enough participants for direct comparison analysis (Figure 5, A and B and Supplemental Figure 22). These 5 responders were further analyzed for E2 effects. While 3 participants showed decreased αCD3/CD28–mediated activation with E2 treatment, one did not change and one increased (Figure 5C). Similar results were seen in the cell lysates (Figure 5D). Since we saw limited activity from IL-15 in this assay, we wanted to confirm that there was no influence from the CSS media on IL-15 activity in CD4^+^ T cells. To determine this, pSTAT5 activation was measured by flow cytometry in male and female donors in total CD4^+^ T cells plated in CSS media and treated with 100 ng/mL IL-15 overnight. No differences were observed between regular RPMI media and CSS media on IL-15–mediated pSTAT5 induction (Supplemental Figure 23).

## Discussion

In this study, we examined the potential effect of biological sex, age, and sex hormones on the ability of IL-15 to promote immune activation and to reactivate latent HIV. It is important to consider these factors when designing HIV cure therapies and clinical trials involving IL-15 or the IL-15 superagonist N-803. We did not observe any effects from sex hormones 17β-estradiol, progesterone, or testosterone derivative danazol on IL-15–mediated immune activation of CD4^+^ T, CD8^+^ T, and NK cells or cytokine secretion. We did observe that IL-15 mediated higher activation of CD4^+^ T cells in female donors than in male donors, and this difference was abrogated at the highest level of E2 treatment. This level of E2 would most likely be seen only during certain stages of pregnancy, during which there are other changes in immune function (34–37, 57). To try to elucidate the mechanism behind this observed sex difference, we analyzed IL-15–mediated pSTAT5 in CD4^+^ T cells from male and female donors but did not see any significant differences (Supplemental Figure 2). This suggests that a possible mechanism for this difference may lie in other IL-15–activated pathways such as through MAPK or PI3K/AKT signaling or other STAT proteins (44). IL-15 has been shown to up- and downregulate many different genes related to proliferation and activation of CD4^+^ T cells, a phenomenon that has not yet been fully investigated with relation to biological sex (58).

We found a positive correlation with age in IL-15–mediated CD8^+^ T cell activation and IFN-γ production. It is known that IL-15 enhances memory CD8^+^ T cell cytotoxicity and increases IFN-γ production (59, 60). A previous study found both higher T cell activation and increased IFN-γ production in older populations with PHA treatment (61). Immune response changes with aging, with increased chronic, low-level inflammation (known as inflamm-ageing), a decline in naive T cells, and an increase in T_TD_ cells (46–48). It is not clear what the effect is on T cell activation or on IFN-γ production (62). However, studies have shown an increase with aging in the population of CD8^+^CD28^–^CD57^+^ effector memory cells that produce IFN-γ (63, 64). This CD8^+^ T cell subset could have an important role in immune response to HIV (64, 65). More studies are needed in understanding immune responses in aging PWH in the context of IL-15 and other cure strategies.

We did not see any effects of E2, P4, biological sex, or age on latency reversal in a primary cell model (54). Interestingly, we observe varying effects from E2 treatment in reactivation of translation-competent reservoirs in total CD4^+^ T cells isolated from PWH who are ART suppressed in our small cohort of participants. This finding in our in vitro model and in PWH contrasts with recent studies showing that ESR-1 regulates latency reversal. A study by Das et al. showed HIV-1 long terminal repeat (LTR) regulation by ESR-1 in Jurkat cells and in a Th17 model of latency, affecting latency reversal (25). Thus far, the mechanism of ESR-1 suppression of HIV-1 transcription and latency regulation has not been fully elucidated. A possible mechanism of ESR-1 regulation of HIV-1 LTR may be partially due to interaction between ESR-1 and β-catenin with subsequent binding to the LTR; however, there is no evidence of direct binding sites for ESR-1 on the HIV-1 LTR (66). Conversely, a recent clinical trial found that tamoxifen, an ESR-1 antagonist, did not impact LRA Vorinostat’s ability to induce expression of HIV RNA in a cohort of women who were postmenopausal and ART-suppressed (67). However, there was only modest latency reversal, which may have limited the ability to observe possible effects from tamoxifen. Though there is evidence of RNA expression of ESR-1 in CD4^+^ T cells, there has been some difficulty in measuring protein expression (49). A study by Pierdominici et al. showed that this may be partially due to differences in antibody sensitivity and specificity (68). In our work, we were able to detect ESR-1 in total CD4^+^ T cells by Western blot only after screening multiple antibodies and then observing expression in only a small number of samples. We were then able to measure ESR-1 by flow cytometry, in which we saw expression in naive, T_CM_, T_TM_, T_EM_, and T_TD_ CD4 subsets. The level of expression was similar among subsets, but we observed a higher expression in T_CM_ CD4 cells. ESR-1 has several isoforms, with 2 truncated N-terminus versions, ERα46 and ERα36 (69). The Pierdominici et al. study suggests that isoform ERα46 may be more prevalent in lymphocytes than the full form, thus potentially limiting the ability of antibodies that recognize the N-terminus region to detect some of these isoforms (68). As such, the evaluation of the expression and function of each isoform in T cells required further evaluation (69). Differences in ESR-1 expression among individuals and the prevalence of different isoforms and their expression on CD4^+^ T cell subsets may all contribute to differences observed in the effects of E2 in different latency models and potentially in reservoirs of latently infected cells from PWH. More studies to find the mechanism of ESR-1 regulation on HIV-1 latency, and how much this may impact HIV cure strategies, are critical. Future studies should also analyze ESR-β, as it has been shown to have potential effects on the immune system and on neurological disorders, including for treatment of HIV-associated neurocognitive disorders (70, 71). Additionally, a study by Devadas et al. showed that high levels of E2 and P4 treatment of macrophages in vitro was correlated with down regulation of HIV-1 replication (72). Thus, further investigation is required on the potential expression of PR and effects from P4 on CD4^+^ T cells and macrophages both directly and indirectly.

Our study has some limitations. First, we did not utilize testosterone, and instead focused on its derivative danazol. Danazol has a broader spectrum of action than testosterone and can alter estrogen, progesterone, glucocorticoid, and gonadotropin signaling pathways in addition to acting as an androgen. Second, the in vitro model used in this study is specifically focused on central memory CD4^+^ T cells, and our data indicates that the impact of ESR-1 regulation on HIV transcription could be subset dependent. The Das et al. study utilized a model derived from our T_CM_ model that differentiates into Th17 cells and reported a direct effect from E2 (25). The main transcription factor of Th17 cells is *RORgT*, which has been shown to be inhibited by E2 through estrogen response elements on the promotor region. Additionally, E2 was shown to affect Th17 differentiation and function (73). This may be a factor in the differences seen with E2 treatment between primary cell models of latency (25). Third, although we saw LRA activity of IL-15 in a primary cell model, we did not observe the same in cells isolated from PWH. This contrasts with previous studies showing the ability of IL-15 and the superagonist N-803 to reactivate latent HIV in CD4^+^ T cells isolated from participants who were ART suppressed (16). It is worth noting that the characteristics of the participants used in this prior study are unknown. Our study, albeit small, included both male and female participants. Additionally, our small cohort of PWH was primarily African American. It will be important to further evaluate diverse populations of PWH when evaluating the activity of different LRAs. It is plausible that different LRAs could have different effects in diverse populations. Increasing our knowledge of the biological factors influencing the activity of LRAs could help in tailoring curative strategies to specific individuals or populations.

In conclusion, more studies are needed to elucidate the potential effects of sex hormones on HIV-1 cure strategies, including the effects of sex hormone–based therapies, contraception, and hormone status with age in diverse populations. There remains a substantial paucity in HIV-1 clinical studies that consider biological sex and age. In particular, women and older individuals are not currently being reflected in these clinical studies at the same rate as the HIV-1 burden they carry (74). Our data point to the need for further research including biological sex and age as critical considerations for HIV cure strategies.

## Methods

### Sex as a biological variable.

All experiments used an equal number of males and females to examine differences by biological sex.

### Reagents.

rhIL-2 and rhIL-15 were provided by the BRB/NCI Preclinical Repository. The following reagent was obtained through the NIH HIV Reagent Program, Division of AIDS, NIAID, NIH: Nelfinavir, Raltegravir, and Human Immunodeficiency Virus 1 (HIV-1), Strain NL4-3 Infectious Molecular Clone (pNL4-3), ARP-114, contributed by M. Martin (75). Sex hormones were obtained from Cayman Chemical Company: 17β-estradiol (cat#10006315), Progesterone (cat#15876), and Danazol (cat#16471). MTT (3-(4,5-Dimethylthiazol-2-yl)-2,5-Diphenyltetrazolium Bromide) (cat#M6494) and Dynabeads Human T-Activator CD3/CD28 (cat#11131D) were purchased from Thermo Fisher Scientific. T47D cell line was gifted from Rong Li at The George Washington University (Washington, DC, USA).

### PBMCs.

Buffy coats were obtained from HIV-negative donors aged 17 years and older from the Gulf Coast Regional Blood Center or frozen PBMCs from STEMCELL Technologies (cat#70025) (Supplemental Table 1). PBMCs were isolated from buffy coats by Lymphoprep cell gradient centrifugation (STEMCELL Technologies, cat#07851). After washing 2 times in PBS + EDTA (2mM), the PBMCs were resuspended in RPMI 1640 medium without phenol red (Cytivia) supplemented with 10% charcoal-stripped FBS (Gibco), 1% L-glutamine, and 1% Penicillin/Streptomycin (Gibco) (CSS media). For Western blot and flow cytometry analysis of subsets, cells were isolated using STEMCELL negative isolation kits for total CD4^+^ T (cat#19052), naive CD4^+^ T (cat#19555), memory CD4^+^ T (cat#19157), total CD8^+^ T (cat#17953), and NK cells (cat#19055).

### Immune activation.

HIV-negative PBMCs were cultured at 3 × 10^6^/mL overnight in RPMI without phenol red and charcoal stripped serum (CSS media) with treatment of 17β-estradiol (0.05, 0.5, and 5 ng/mL), progesterone (0.25, 2.5, 25 ng/mL), or danazol (0.3, 3, 30 ng/mL). Then IL-15 was added at 100 ng/mL for 48 hours. 1 × 10^6^ cells were collected and stained for flow cytometry analysis and supernatants were frozen at –20°C for cytokine analysis.

### Cytokine analysis.

Frozen supernatants were thawed, and the assay was performed according to Quanterix manufacturer protocol. Briefly, samples were incubated on the 96-well prespotted plate for 2 hours at room temperature (RT) shaking at 525 rpm. Then plate was washed with Quanterix wash buffer (cat#1863332) using a plate washing machine and then incubated first with biotinylated antibody and then with streptavidin-HRP for 30 minutes each at RT shaking at 525 rpm. Plates were washed between each step. After the final incubation, the plate was washed with a 5-step wash, then SuperSignal was added from the kit and the plate was read. Ten cytokines were measured using Quanterix SP-X Corplex Cytokine Panel (IFN- γ, IL-1β, IL-4, IL-5, IL-6, IL-8, IL-10, IL-12p70, IL-22, and TNF-α) (product# 85-0329).

### Granzyme B.

Frozen supernatants were thawed, and granzyme B levels were evaluated using U-PLEX Human Granzyme B Assay (cat#K151APDK-1) according to MSD manufacturer protocol. Briefly, biotinylated capture antibody was incubated on MSD GOLD Small Spot Streptavidin Plate with shaking for 1 hour at RT. Plate was washed with PBS-T. Calibrators and samples are incubated with shaking for 2 hours at RT. The plate was washed and detection antibody was added and incubated with shaking for 1 hour. After the final wash, MSD GOLD Read Buffer B was added to wells and plate was read.

### MTT assay.

T47D breast cancer cells were preconditioned for 7 days in either complete RPMI media or CSS media. Cells were then plated and incubated for 72 hours in either RPMI media control or CSS media with 0.05 ng/mL, 0.5 ng/mL, or 5n g/mL E2 and a no E2 control. CyQUANT MTT Cell Proliferation Assay Kit rapid protocol was performed according to manufacturer’s instructions.

### Proliferation assay.

Total CD4^+^ T cells were isolated with STEMCELL negative isolation kits for total CD4^+^ T (cat#19052). Cells were treated with CellTrace Yellow (Thermo Fisher Scientific, cat#C34573) using 1 μL of dye per mL of cells and incubated at 37°C for 20 minutes. Cells were washed to remove excess dye and then resuspended at 1 × 10^6^/mL in CSS media and pretreated for 2 hours with and without hormones. IL-15 was added (100 ng/mL) and then cells were incubated for 7 days, after which cells were stained (see *Proliferation* below).

### Flow cytometry.

To assess CD4 surface expression (APC, clone S3.5, Thermo Fisher Scientific, cat# MHCD0405) and intracellular p24-Gag expression (FITC, clone KC57, Beckman Coulter, cat#6604665), 2.5 × 10^5^ cells were fixed, permeabilized, and stained as previously described (76). All experiments were run on a BD LSR Fortessa X20 flow cytometer with FACSDiva software (Becton Dickinson). Data were analyzed using FlowJo (BD Biosciences).

### Immune activation.

1 × 10^6^ cells were washed with PBS, incubated with human Fc block (cat#564220, BD Biosciences) for 10 minutes at RT, and then cells were stained with the surface markers CD3 BV786 (clone SP34-2, cat#563800, BD Biosciences), CD4 FITC (clone RPA-T4, cat#300538, BioLegend), CD8 PE (clone OKT8, Thermo Fisher Scientific, cat#12-0086-42), CD56 PerCP/Cy5.5 (clone 5.1H11, BioLegend, cat#362506), CD69 APC-Cy7 (clone FN50, BioLegend, cat#310914), and eBioscience Fixable Viability Dye eFluor 450 (Thermo Fisher Scientific, cat#65-0863-18) in FACS buffer (PBS + 2% FBS) for 30 minutes at 4°C. Then cells were washed and resuspended in 3% PFA. All experiments were run on a BD LSR Fortessa X20 flow cytometer with FACSDiva software. Data were analyzed using FlowJo.

### ESR-1 analysis in CD4 T cells.

1 × 10^6^ cells were washed and stained with the surface markers CD4 BV711 (clone RPA-T4, BD Biosciences, cat#568371), CCR7 APC-eFlour 780 (clone 3D12, Thermo Fisher Scientific, cat#47-1979-42), CD27 FTIC (clone O323, Thermo Fisher Scientific, cat#11-0279-42), CD45RA PerCP-Cy5.5 (clone HI100, Thermo Fisher Scientific, cat#45-0458-42), and CD45RO PE (clone UCHL1, BioLegend, cat#304206) for 30 minutes at 4°C. Cells were washed with FACS and then resuspended in BD Cytofix/Cytoperm (cat# 554722) for 30 minutes at 4°C. Cells were washed with BD Perm/Wash buffer (cat# 554723) and then incubated with anti-ESR-1 Alexa Fluor 647 (clone SP1, Abcam, cat#ab267512) or anti-IgG isotype control Alexa Fluor 647 (clone EPR25A, Abcam, cat#ab199093) for 30 minutes at 4°C. For analysis with IL-15, after isolation, cells were plated in CSS media at 1 × 10^6^ and treated with 100 ng/mL of IL-15 overnight. Then cells were stained as above. All experiments were run on spectral flow cytometer Cytek Aurora analyzer. Data was analyzed using FlowJo.

### Proliferation.

5 × 10^5^ cells were washed with PBS and stained with viability dye (eBioscience Fixable Viability Dye eFluor 450, Thermo Fisher Scientific, cat#65-0863-18), CD4 APC (clone OKT4, Thermo Fisher Scientific, cat#17-0048-42), and CD45RO BV786 (clone UCHL1, BD Bioscience, cat#564290) for 30 minutes at 4°C and then washed and resuspended in 3% PFA. CellTrace Yellow was visualized by PE. All experiments were run on a BD Celesta analyzer flow cytometer with FACSDiva software. Data were analyzed using FlowJo.

### pSTAT5 analysis in CD4^+^ T cells.

5 × 10^5^ cells were washed with PBS and resuspended in 100 mL FACS buffer with viability dye (eBioscience Fixable Viability Dye eFluor 450, Thermo Fisher Scientific, cat#65-0863-18). Cells were incubated for 10 minutes at 4°C and washed with 1 mL FACS buffer. Cells were then resuspended in 100 μL prewarmed Fix Buffer I (BD Bioscience, cat#557870) and incubated at 37°C for 10 minutes. Cells were washed with 1 mL FACS buffer, resuspended in 200 μL of precooled Perm Buffer III (BD Bioscience, cat#558050) added dropwise while vortexing and then incubated on ice for 30 minutes. Cells were washed with 1 mL FACS buffer then resuspended with 100 μL FACS buffer with 2.5 mL pSTAT5(Y694)-PE (clone A17016B.Rec, BioLegend, cat#936903) and were then incubated for 1 hour at RT in the dark. Then cells were washed with 1 mL FACS buffer and resuspended in 200 μL PBS + 2% PFA. All experiments were run on a BD LSR Fortessa X20 flow cytometer with FACSDiva software. Data were analyzed using FlowJo.

### Western blots.

Untreated cells were isolated (kits listed below) and between 5–10 × 10^6^ cells were then washed with 1 mL PBS. Cells were then lysed with NETN extract buffer containing 100 mM NaCl, 20 mM Tris-Cl (pH 8), 0.5 mM EDTA, 0.5% Nonidet P-40, protease inhibitor cocktail (cOmplete; Roche), and phosphatase inhibitor cocktail (phosSTOP; Roche) for 30 minutes on ice. Lysates were cleared by centrifugation at 13,200*g* for 10 minutes at 4°C. Proteins were visualized by SDS-PAGE. Western blotting was performed according to standard protocols. Briefly, 10 μg of sample protein with NETN cell lysis buffer up to 20 μl was mixed with 4 μl of 6× Laemelli buffer to make 24 μl total sample to load into the gel. The samples were heated at 100°C for 5 minutes and then spun down at 13,200*g* for 1 minute. The gel was run at 90V until samples were within the gel then voltage was adjusted to 120V. PVDF membranes are activated with 100% methanol. Transfer was done in the semi-dry BioRad Trans-blot system. Following transfer, the membrane was washed in PBS with 1% Tween20 and blocked in blocking buffer (5% milk in PBS) for 2 hours. The membrane was then washed again, and primary antibody was added with incubation buffer (2% milk in PBS) and incubated overnight at 4°C. Secondary was incubated the following day for 2 hours at RT. The blot was imaged with Immobilon Western Chemiluminescent HRP Substrate (cat#WBKLS0500). The following antibodies were used at the following concentrations: anti-β-actin antibody at a 1:10,000 dilution (clone AC-15, Sigma-Aldrich, cat#A5441); GAPDH at a 1:10,000 dilution (clone D16H11, Cell Signaling Technology, cat#5174S);and secondary polyclonal anti-rabbit and anti-mouse antibodies (Jackson ImmunoResearch, cat#111-035-046 and 115-035-146, respectively) were used at a 1:5,000 dilution and 1:10,000 dilution, respectively. Estrogen receptor α (clone D8H8, Cell Signaling Technology, cat#8644S) at 1:5,000 dilution; Estrogen receptor α (clone EPR4097, Abcam, ab108398); androgen receptor (clone AR441, Thermo Fisher Scientific, cat#MA5-13426); and progesterone receptor (clone hPRa2, Thermo Fisher Scientific, cat#MA5-12642) at 1:5,000 dilution for Supplemental Figure 18. For analysis of cell subsets for sex hormone receptor expression, cells were isolated using STEMCELL negative isolation kits for total CD4^+^ T (cat#19052), naive CD4^+^ T (cat#19555), memory CD4^+^ T (cat#19157), total CD8^+^ T (cat#17953), and NK cells (cat#19055).

### Model of latency.

Naive CD4^+^ T cells were isolated using the EasySep Human naive CD4^+^ T Cell Enrichment Kit (Stem Cell, cat#19555) according to the manufacturer’s instructions. The T_CM_ model of latency was performed as previously described using NL4-3 to generate latently infected CD4 central memory cells (54, 55). Briefly, naive CD4^+^ T cells were activated at 0.5 × 10^6^ cells/mL with αCD3/CD28 Dynabeads in the presence of αIL-4, αIL-12, and TGF-β in RPMI supplemented with 1% L-Glutamine, 10% FBS, and 1% penicillin/streptomycin (complete RPMI) for 3 days. On day 3, Dynabeads were removed and cells were cultured in complete RPMI with IL-2, which is used throughout. On day 7, cells were infected by spinoculation and then on day 10, cells were “crowded” in 96-well round bottom plates to facilitate spread of infection. On day 13, the cells were uncrowded and plated in the presence of ART (1 μM Raltegravir + 0.5μM Nelfinavir). On day 17, CD4^+^ cells were sorted from the infected cultures by positive selection (Dynabeads CD4 positive Isolation kit, Thermo Fisher Scientific #11331D). This model was modified on day 17 of the timeline, cells were cultured in CSS media. Cells were then pretreated with hormones 17β-estradiol (0.05, 0.5, and 5 ng/mL) or progesterone (0.25, 2.5, 25 ng/mL) for 2 hours, in the presence of ART (1 μM Raltegravir + 0.5 μM Nelfinavir), before adding LRAs, either IL-15 (100 ng/mL) or αCD3/CD28 according to manufacture’s protocol. Reactivation was measured by analyzing intracellular p24 expression by flow cytometry on day 19. See Supplemental Figure 24 for the timeline.

### Participant samples from PWH.

Blood samples were obtained from PWH who were ART suppressed at the George Washington Medical Faculty Associates. Participants include people who were HIV positive who were 18 years or older, have been on ART for at least 24 months, have undetectable viral load (< 50 copies/mL by current assays in place) for at least 6 months, CD4^+^ cell count > 350 cells/mm^3^, and capable of giving informed consent and consent to have their clinical information on the HIV data registry (Supplemental Table 3). PBMCs are isolated from blood and total CD4^+^ T cells are isolated using negative isolation kit (StemCell, cat#19052). Cells were counted and resuspended in CSS media at a concentration of 750,000 cells/150 μL with ART (1 μM Raltegravir + 0.5 μM Nelfinavir) and plated in a 96-well round bottom plate with or without 300 pg/mL E2 for 2 hours and then incubated with IL-15 (100 ng/mL) or αCD3/CD28 for 96 hours. After 96 hours, αCD3/CD28 wells were resuspended, placed on a plate magnet for 1 minute, and suspensions were moved to a new row to remove beads. Plates were then spun down at 443*g* for 5 minutes at 4°C and supernatants were moved to a fresh plate. Then cell pellets were lysed using 75 μL NETN (see Western blot section). Plates were sealed with plate covers and stored at –80°C until analysis. Prior to p24 analysis, 1% triton was added to supernatants to ensure viral inactivation. Lysates were thawed on ice for 30 minutes and spun down at 886*g* for 30 minutes at 4°C. Levels of p24 in supernatants and lysates were measured as previously described by p24 ultra-sensitive ELISA (Quanterix Homebrew product#100-0461) (56).

### Statistics.

Statistical analyses were performed using GraphPad Prism 9.4.1 software. Experiments were analyzed by Wilcoxon paired, nonparametric (comparing agonist treated to untreated), Kruskal-Wallis (comparing hormone treatment groups), Mann-Whitney unpaired, nonparametric test (comparing biological sex), and Spearman’s correlation tests (age analysis). A *P* value less 0.05 was considered significant. All the data with error bars are presented as mean values ± SD. ROUT outlier test was used to remove outliers. The statistical analysis used is indicated in each figure legend.

### Study approval.

PWH were recruited from the George Washington Medical Faculty Associates, through a protocol approved by the George Washington University Institutional Review Board (IRB021848). All participants were adults and gave written informed consent prior to their participation.

### Data and materials availability.

All data are available in the main text, the supplementary materials, or the Supporting Data Values file.

## Author contributions

Work was conceptualized by AB and CSH. CSH, CL, and AB developed the methodology. Investigation was performed by CSH, CL, and MA. CSH and AB were responsible for visualization. AB and CSH were responsible for funding acquisition. AB was the project administrator and supervisor. MS provided clinical samples. MG, NMA, and CC provided advice and expertise. CSH and AB wrote the original manuscript draft. CSH, AB, MG, MS, NA, and CC reviewed and CSH and AB edited the manuscript. All the authors approved the final version.

## Supplementary Material

Supplemental data

Unedited blot and gel images

Supporting data values

## Version 1 07/30/2024

In-Press Preview

## Version 2 09/10/2024

Electronic publication
