# Supplementary material for: HIV-1 latency reversal and immune enhancing activity of IL-15 is not influenced by sex hormones
Source: JCI Insight. 2024 Sep 10;9(17):e180609. doi: 10.1172/jci.insight.180609 (PMC11389825; doi:10.1172/jci.insight.180609)
Supplement: Supplemental data [file jciinsight-9-180609-s006.pdf]

Supplementary Materials for

HIV-1 latency reversal and immune enhancing activity of IL-15 is not influenced by sex hormones.

Carissa S. Holmberg, Callie Levinger, Marie Abongwa, Cristina Ceriani, Nancie Archin, Marc Siegel, Mimi Ghosh, and Alberto Bosque

Corresponding author: abosque@gwu.edu

The PDF file includes:

Tables 1 to 3

Figures S1 to S15

Supplemental Figures

Donor ID	Symbol	Source	Biological sex	Age	Race/ethnicity
D001	Circle	STEMCELL	Female	17	Mixed
D002	Asterisk	STEMCELL	Male	30	Caucasian
D003	Circle	STEMCELL	Male	32	Caucasian
D004	Square	STEMCELL	Female	26	Asian
D005	Diamond	STEMCELL	Female	36	Caucasian
D006	Square	STEMCELL	Male	50	Caucasian
D007	Diamond	STEMCELL	Male	43	Mixed
D008	Hexagon	STEMCELL	Female	23	Mixed
D009	Hexagon	STEMCELL	Male	28	Asian
D010	Triangle	STEMCELL	Female	34	Caucasian
D011	Triangle	STEMCELL	Male	23	Caucasian
D012	Inverted Triangle	STEMCELL	Female	43	Caucasian
D013	Inverted Triangle	STEMCELL	Male	19	Caucasian
D014	Square with X	STEMCELL	Female	53	Caucasian
D015	Square with X	STEMCELL	Male	30	Asian
D016	Cross	Gulf Coast Blood Bank	Female	65	N/A
D017	X	Gulf Coast Blood Bank	Female	61	N/A
D018	Asterisk	Gulf Coast Blood Bank	Female	42	N/A
D019	X	Gulf Coast Blood Bank	Male	68	N/A
D020	Cross	Gulf Coast Blood Bank	Male	65	N/A

Table 1. Table of donors for IL-15-mediated immune activation (Figures 1 and 2).

Donor ID	Symbol	Source	Biological sex	Age
D002	Diamonnd	STEMCELL	Male	30
D240	Square with X	Gulf Coast Blood Bank	Male	69
D307	Cross	Gulf Coast Blood Bank	Male	17
D344	Square	Gulf Coast Blood Bank	Male	33
D348	Triangle	Gulf Coast Blood Bank	Male	38
D390	Circle	Gulf Coast Blood Bank	Male	30
D396	Hexagon	Gulf Coast Blood Bank	Male	57
D399	Inverted Triangle	Gulf Coast Blood Bank	Male	63
D403	Circle with X	Gulf Coast Blood Bank	Male	22
D404	Astrisk	Gulf Coast Blood Bank	Male	55
D128	Circle with X	Gulf Coast Blood Bank	Female	68
D320	Square	Gulf Coast Blood Bank	Female	25
D333	Cross	Gulf Coast Blood Bank	Female	33
D341	Triangle	Gulf Coast Blood Bank	Female	18
D349	Inverted Triangle	Gulf Coast Blood Bank	Female	40
D385	Circle	Gulf Coast Blood Bank	Female	40
D398	Square with X	Gulf Coast Blood Bank	Female	17
D400	Hexagon	Gulf Coast Blood Bank	Female	18
D401	Astrisk	Gulf Coast Blood Bank	Female	36
D402	Diamond	Gulf Coast Blood Bank	Female	56

Table 2. Table of donors for the T_{CM} model of latency (Figure 4).

Donor ID	Age	Biological sex	Race/ethnicity	CD4	HIV VL
298S	52	Male	white	497	<20
299S	76	Male	black/AA	730	not detected
301S	50	Female	black/AA	1021	<20
302S	36	Male	black/AA	1523	not detected
303S	52	Male	black/AA	816	not detected
304S	37	Male	black/AA	654	not detected
318S	30	Female	black/AA	1047	not detected
328S	59	Female	black/AA	605	<20
338S	39	Female	black/AA	1151	<20
343S	43	Female	black/AA	806	not detected

Table 3. Table of ART-suppressed people with HIV (Figure 5).

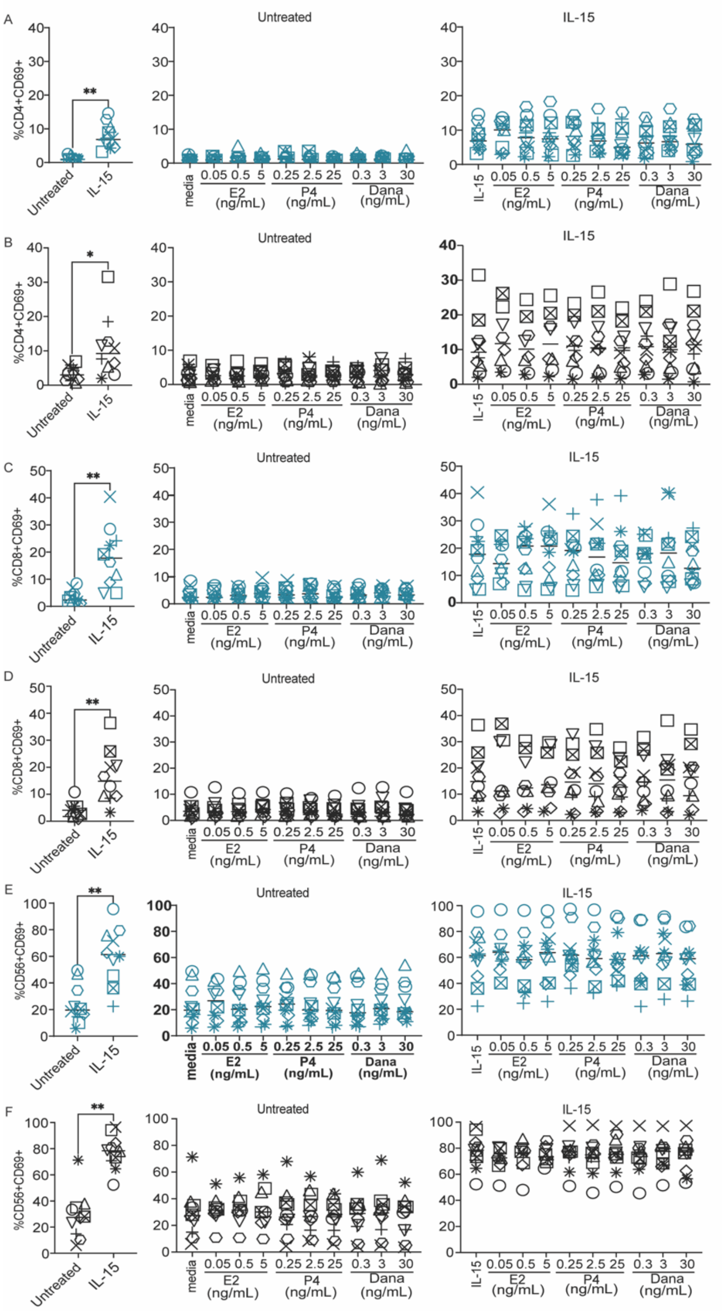

<u>SF1.</u> Immune activation with sex hormone treatment E2, P4, and danazol in CD4 T, CD8 T, and NK cells. (A) Immune activation of CD4 T cells from female donors with IL-15 treatment compared to untreated (left), treatment with E2, P4, and danazol without agonist (middle), and treatment with IL-15 in the presence of E2, P4, and danazol (right). The same conditions are repeated for male CD4 T cells (B), CD8 T cells from female (C) and male donors (D), CD56 cells from female (E) and male donors (F). Female donors are teal symbols and male donors are black symbols. Wilcoxon matched-pairs signed rank test was used to calculate p values (*$p < 0.05$).

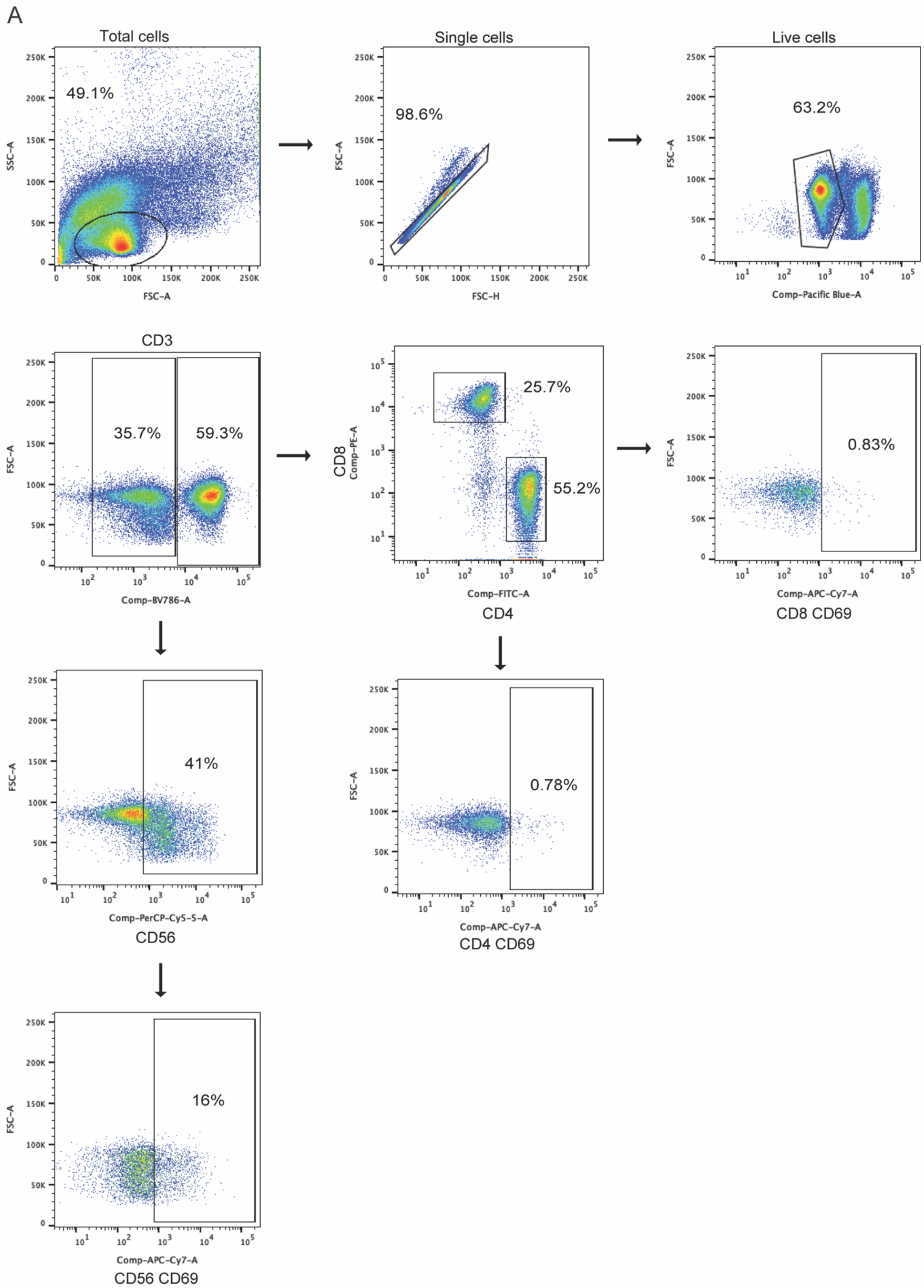

SF2. Representive donor showing gating strategy of untreated PBMCs for immune activation experiment.

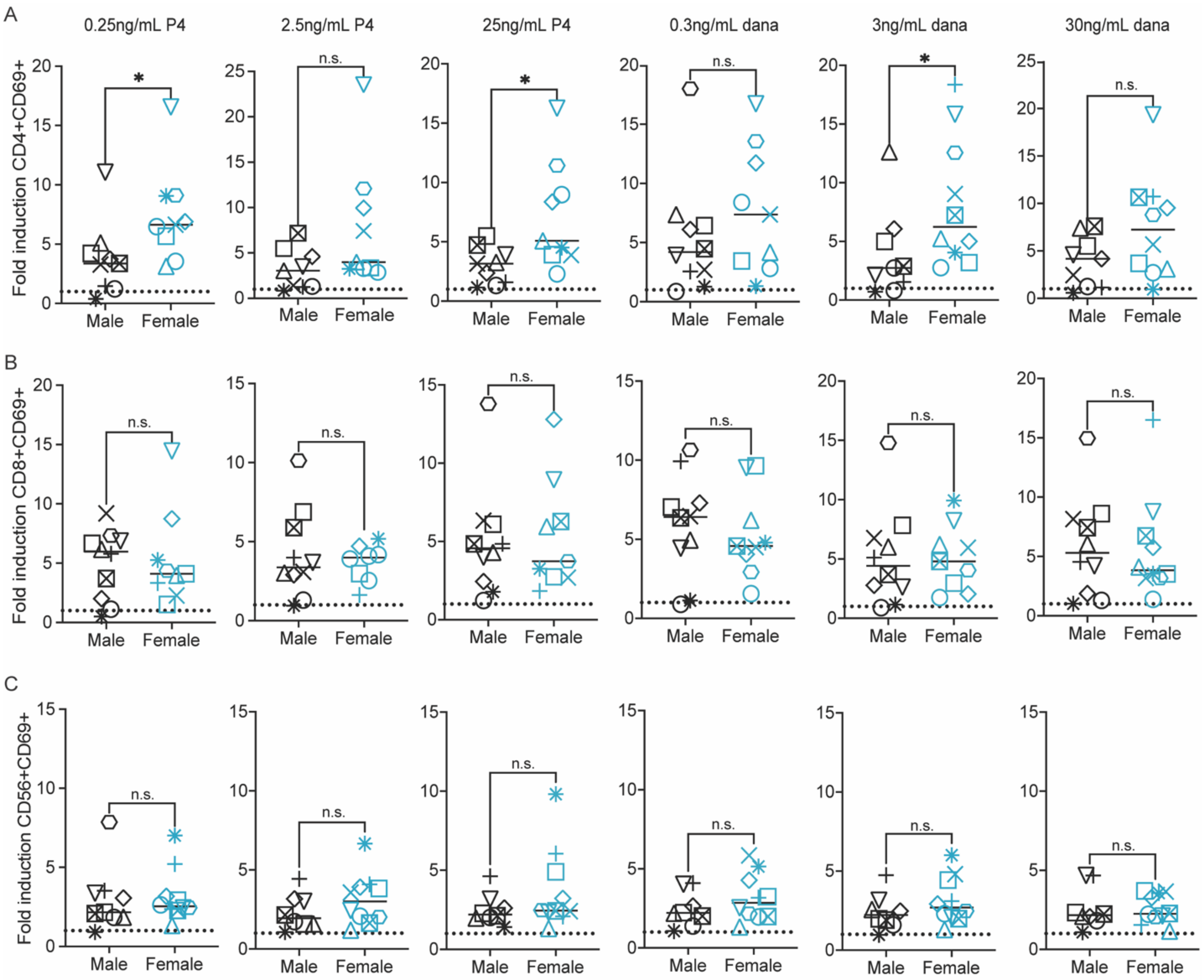

SF3. Biological sex influences IL-15-mediated immune activation of CD4 T cells. Comparative analysis of immune activation mediated by IL-15 (100ng/mL) in 10 age-matched male and female (A) CD4 T cells, (B) CD8 T cells, (C) NK cells in the presence of P4 or dana. Teal symbols are female donors and black symbols are male donors. Mann-Whitney test was used to calculate p values (*$p < 0.05$). ROUT outlier test was used to remove outliers.

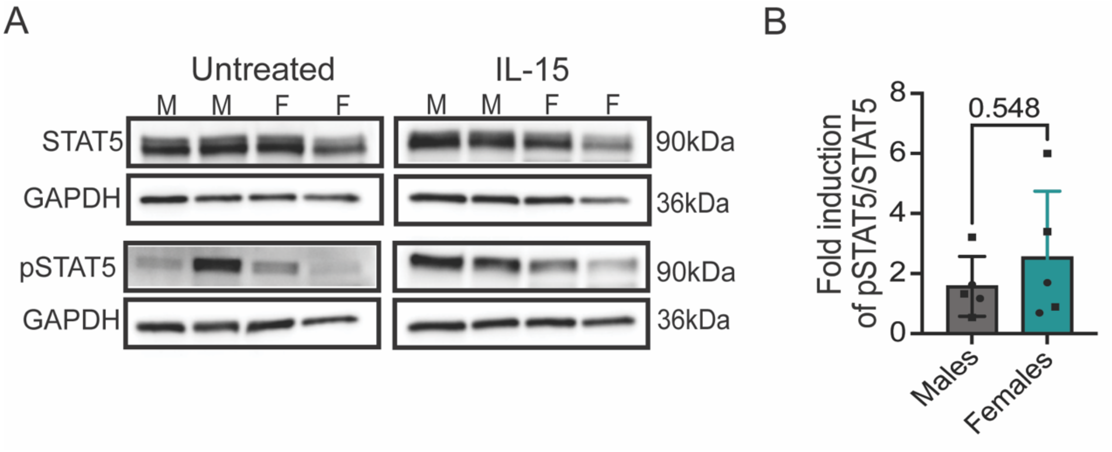

<u>SF4.</u> Western blot analysis of IL-15-mediated STAT5 phosphorylation in total CD4 cells with CSS media. (A) Levels of STAT5 and pSTAT5 in CD4T cells untreated (left) or treated with IL-15 (right) overnight in CSS media. Representative donors (2 males and 2 females). (B) Levels of pSTAT5 over STAT5 after IL-15 treatment in 5 male and 5 female donors.

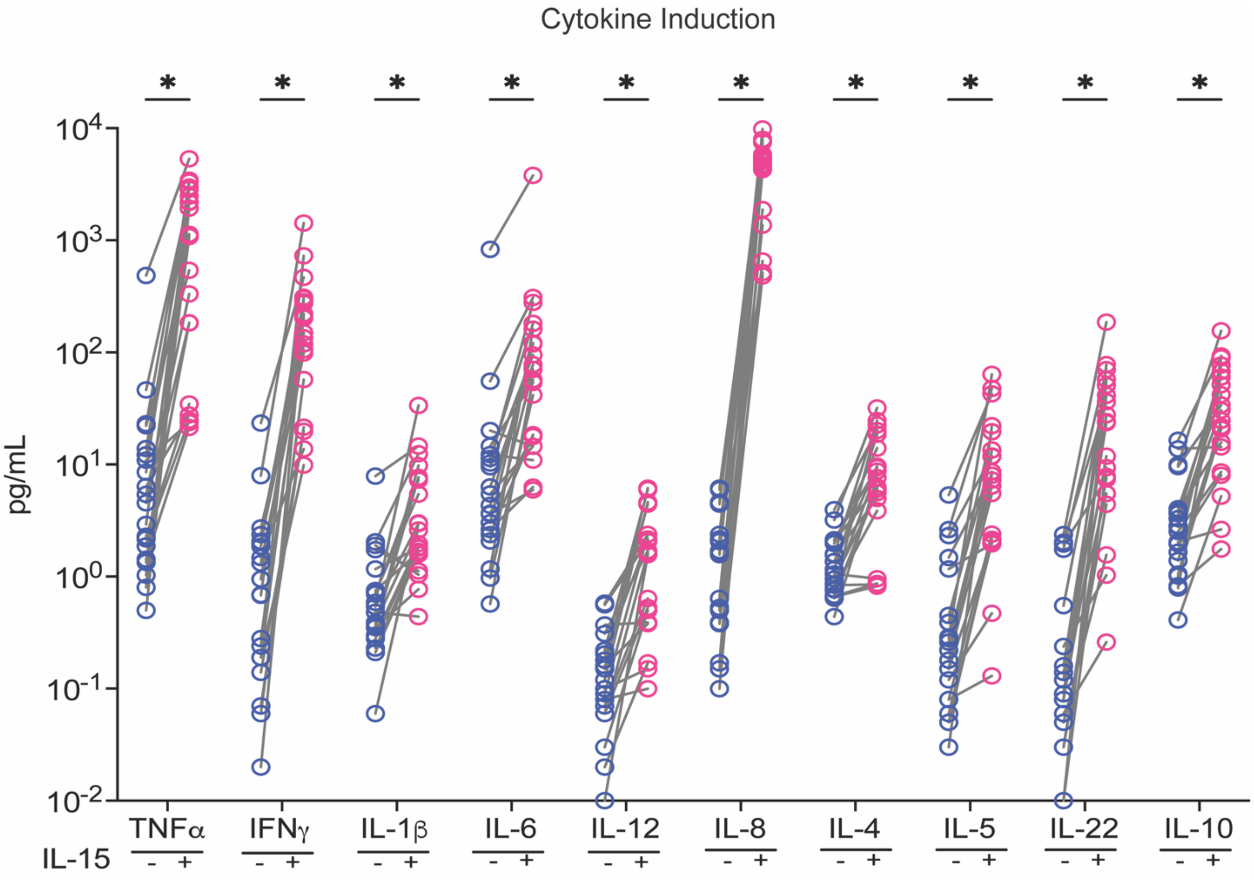

SF5. IL-15-mediated cytokine induction. Levels of each indicated cytokine in unstimulated (blue circles) or IL-15-stimulated PBMCs (pink circles). Multiple Wilcoxon tests was used to calculate significance (****q < 0.0001).

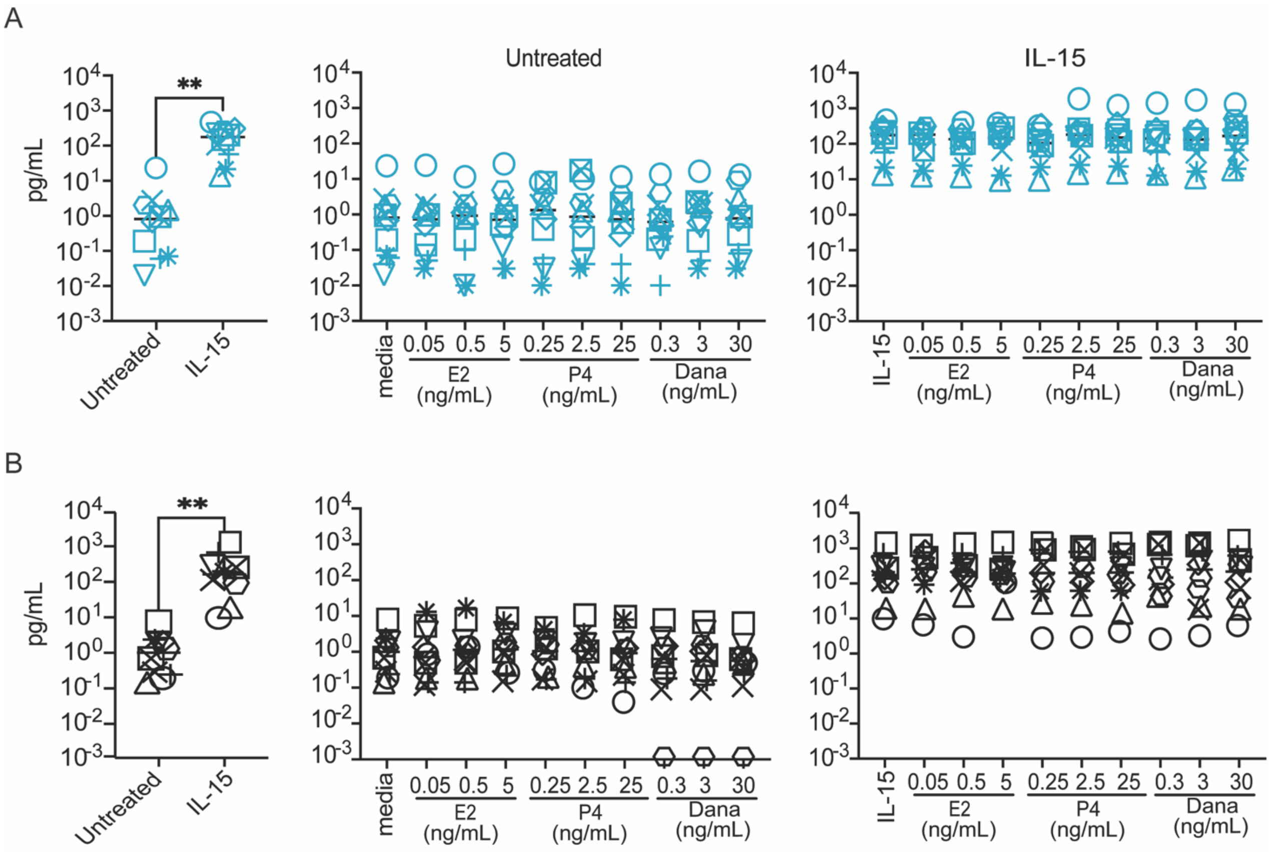

SF6. Analysis of IL-15-mediated IFN-γ induction in PBMCs in the presence of sex hormones. IFN-γ production before and after stimulation with IL-15 (first panel), hormone treatments of estradiol (E2), progesterone (P4), and danazol (dana) alone (middle panel), or in the presence of IL-15 (right panel). Teal symbols are female donors (A) and black symbols are male donors (B). Mann-Whitney test was used to calculate p values (*$p < 0.05$).

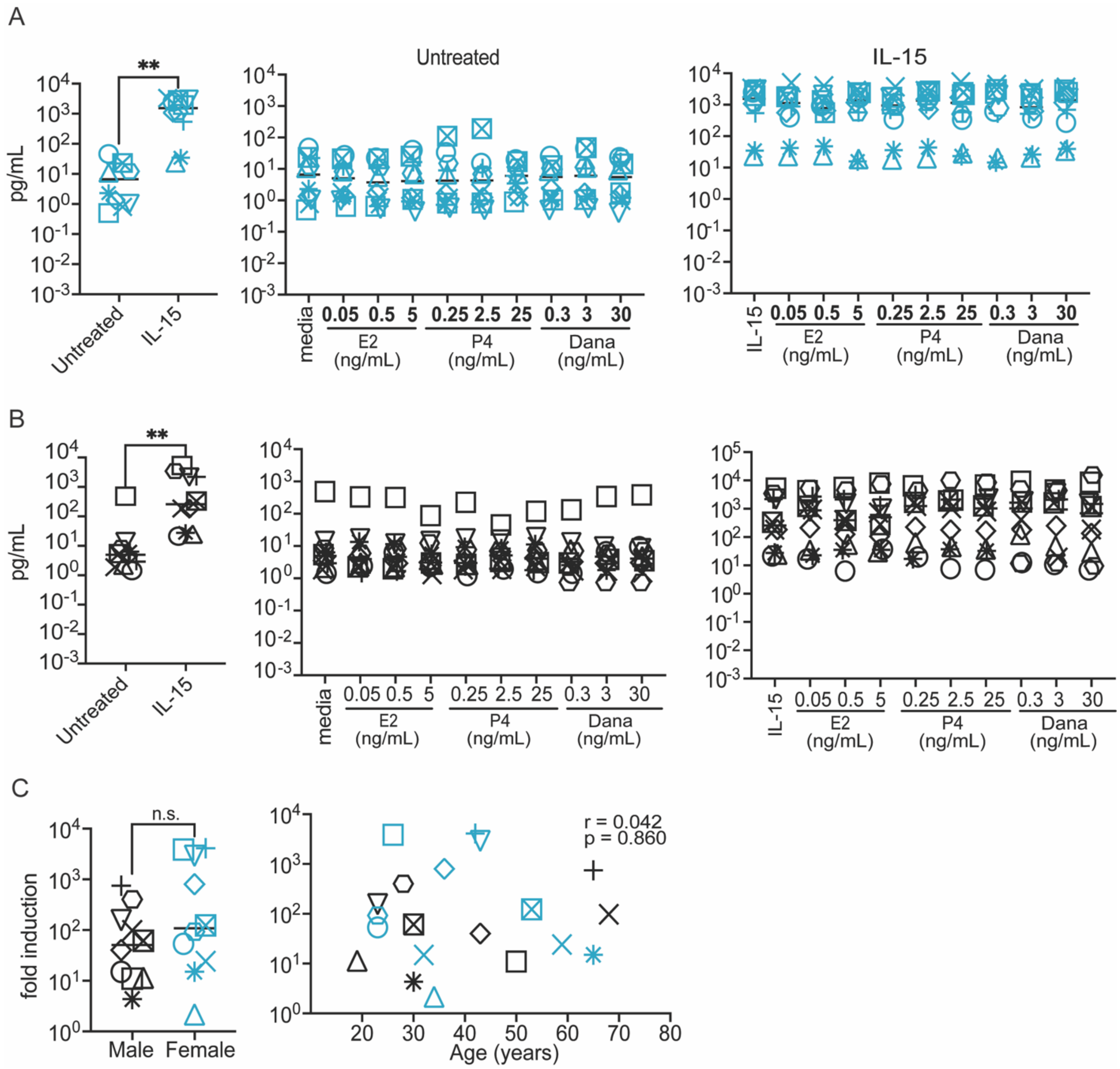

SF7. Analysis of IL-15-mediated TNF-α induction in PBMCs in the presence of sex hormones. TNF-α production before and after stimulation with IL-15 (first panel), hormone treatments of estradiol (E2), progesterone (P4), and danazol (dana) alone (middle panel), or in the presence of IL-15 (right panel). Teal symbols are female donors (A), and black symbols are male donors (B). (C) Fold induction of TNF-α production by biological sex (left) and by age of donor (right). Mann-Whitney test and nonparametric spearman correlation were used to calculate p values (*$p < 0.05$).

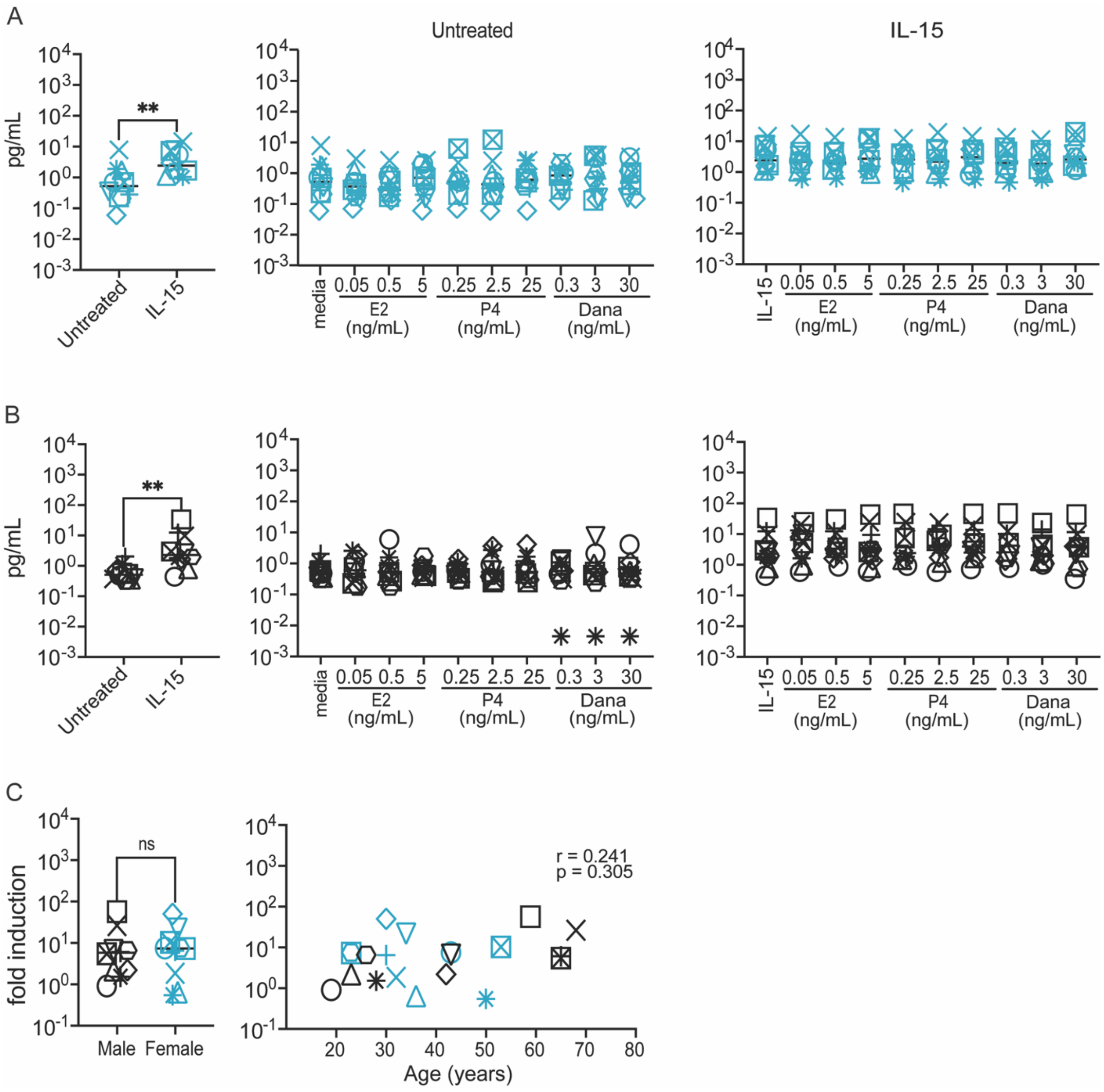

SF8. Analysis of IL-15-mediated IL1-β induction in PBMCs in the presence of sex hormones. IL1-β production before and after stimulation with IL-15 (first panel), hormone treatments of estradiol (E2), progesterone (P4), and danazol (dana) alone (middle panel), or in the presence of IL-15 (right panel). Teal symbols are female donors (A), and black symbols are male donors (B). (C) Fold induction of IL1-β production by biological sex (left) and by age of donor (right). Mann-Whitney test and nonparametric spearman correlation were used to calculate p values (*p < 0.05).

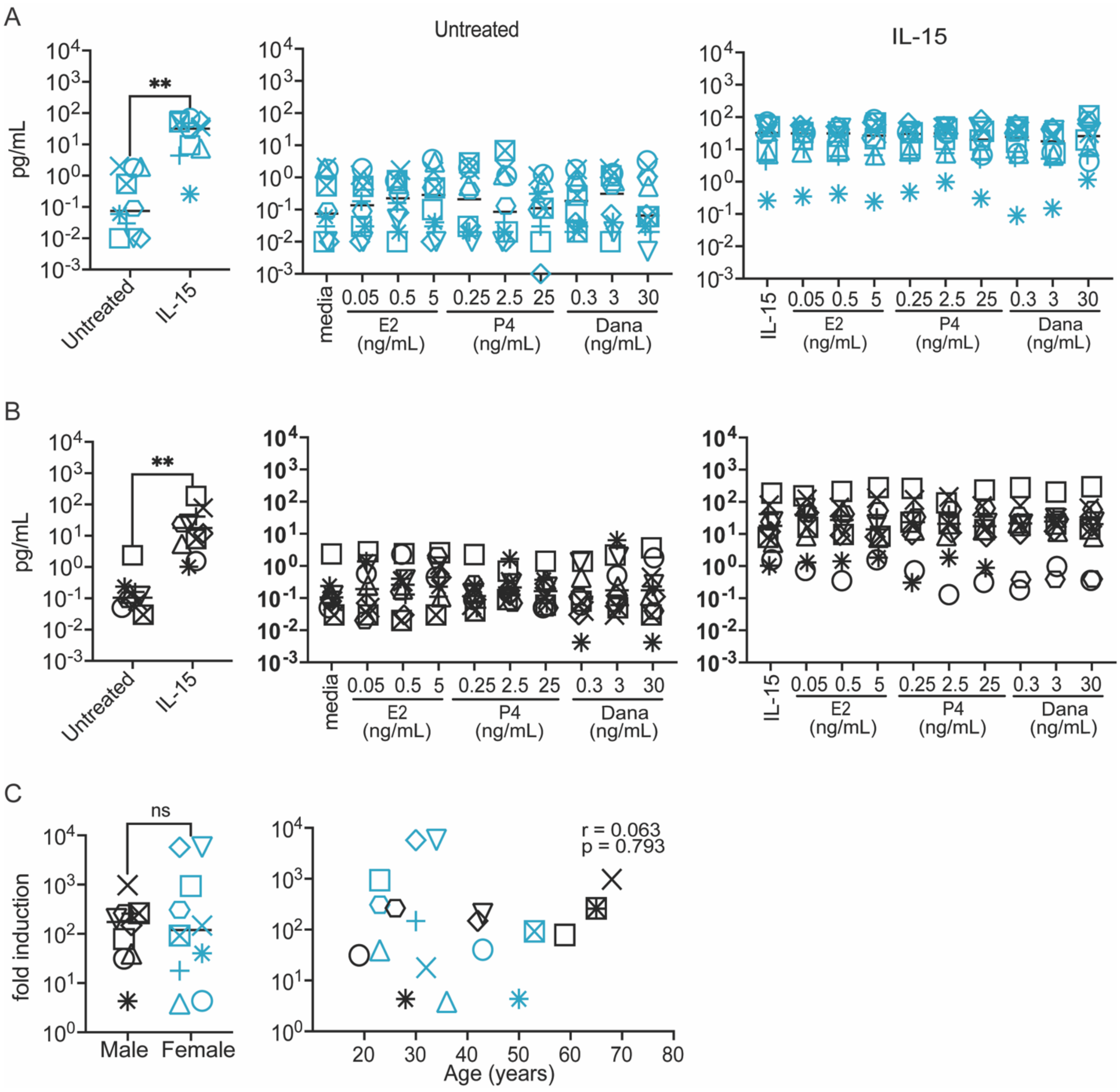

SF9. Analysis of IL-15-mediated IL-22 induction in PBMCs in the presence of sex hormones. IL-22 production before and after stimulation with IL-15 (first panel), hormone treatments of estradiol (E2), progesterone (P4), and danazol (dana) alone (middle panel), or in the presence of IL-15 (right panel). Teal symbols are female donors (A), and black symbols are male donors (B). (C) Fold induction of IL-22 production by biological sex (left) and by age of donor (right). Mann-Whitney test and nonparametric spearman correlation were used to calculate p values (*p < 0.05).

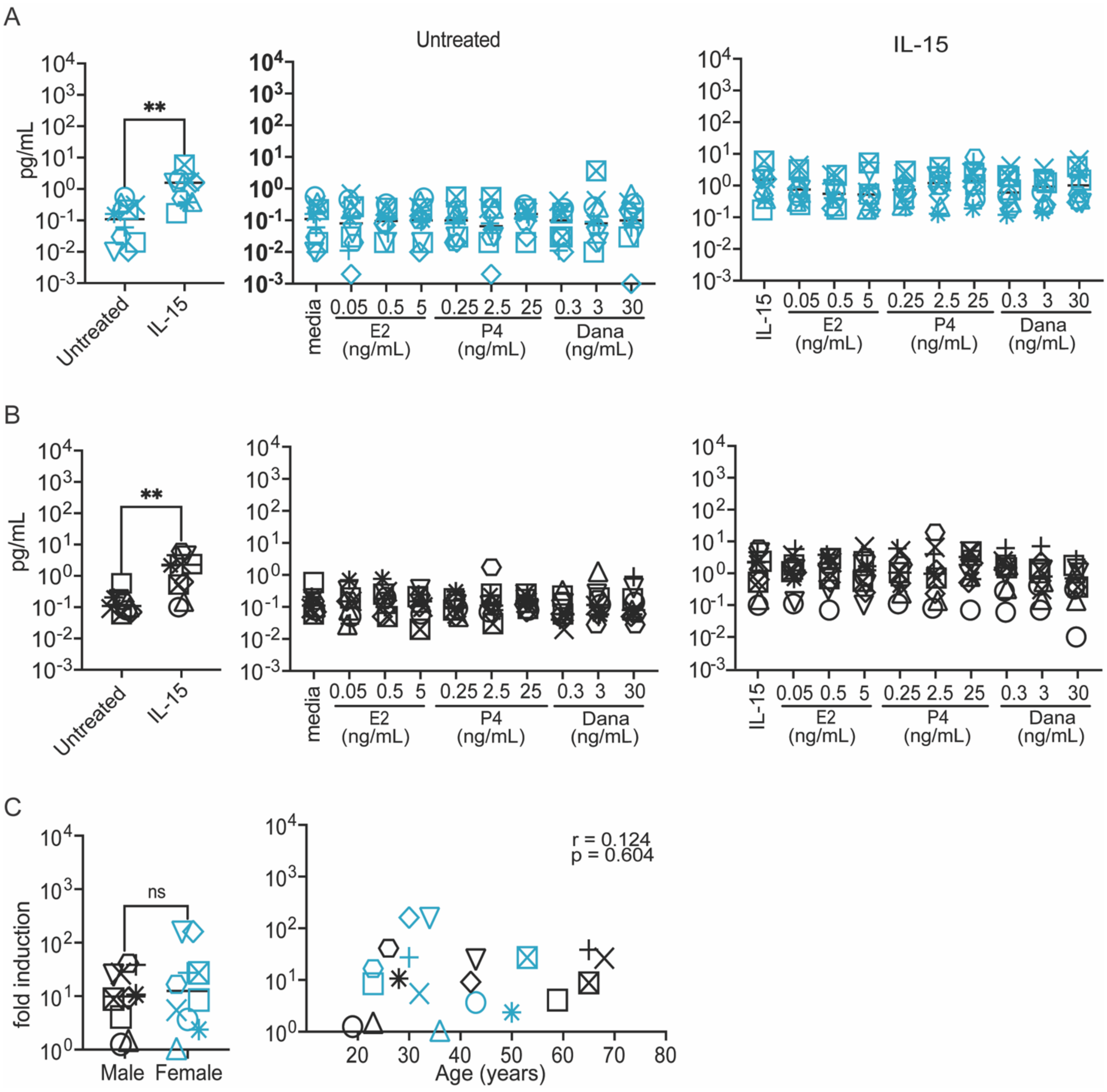

SF10. Analysis of IL-15-mediated IL-12 induction in PBMCs in the presence of sex hormones. IL-12 production before and after stimulation with IL-15 (first panel), hormone treatments of estradiol (E2), progesterone (P4), and danazol (dana) alone (middle panel), or in the presence of IL-15 (right panel). Teal symbols are female donors (A), and black symbols are male donors (B). (C) Fold induction of IL-12 production by biological sex (left) and by age of donor (right). Mann-Whitney test and nonparametric spearman correlation were used to calculate p values (*$p < 0.05$).

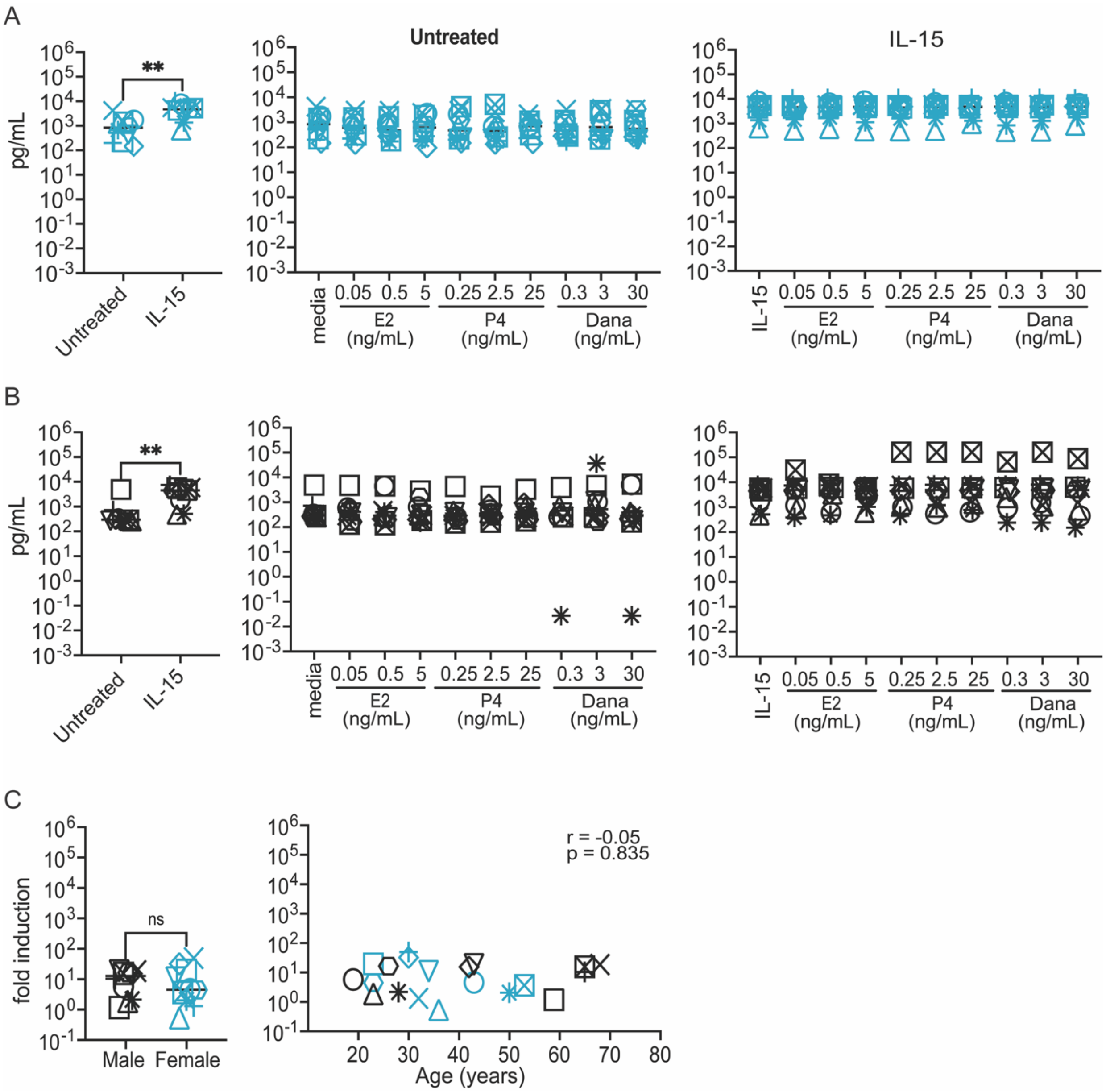

SF11. Analysis of IL-15-mediated IL-8 induction in PBMCs in the presence of sex hormones. IL-8 production before and after stimulation with IL-15 (first panel), hormone treatments of estradiol (E2), progesterone (P4), and danazol (dana) alone (middle panel), or in the presence of IL-15 (right panel). Teal symbols are female donors (A), and black symbols are male donors (B). (C) Fold induction of IL-8 production by biological sex (left) and by age of donor (right). Mann-Whitney test and nonparametric spearman correlation were used to calculate p values (*p < 0.05).

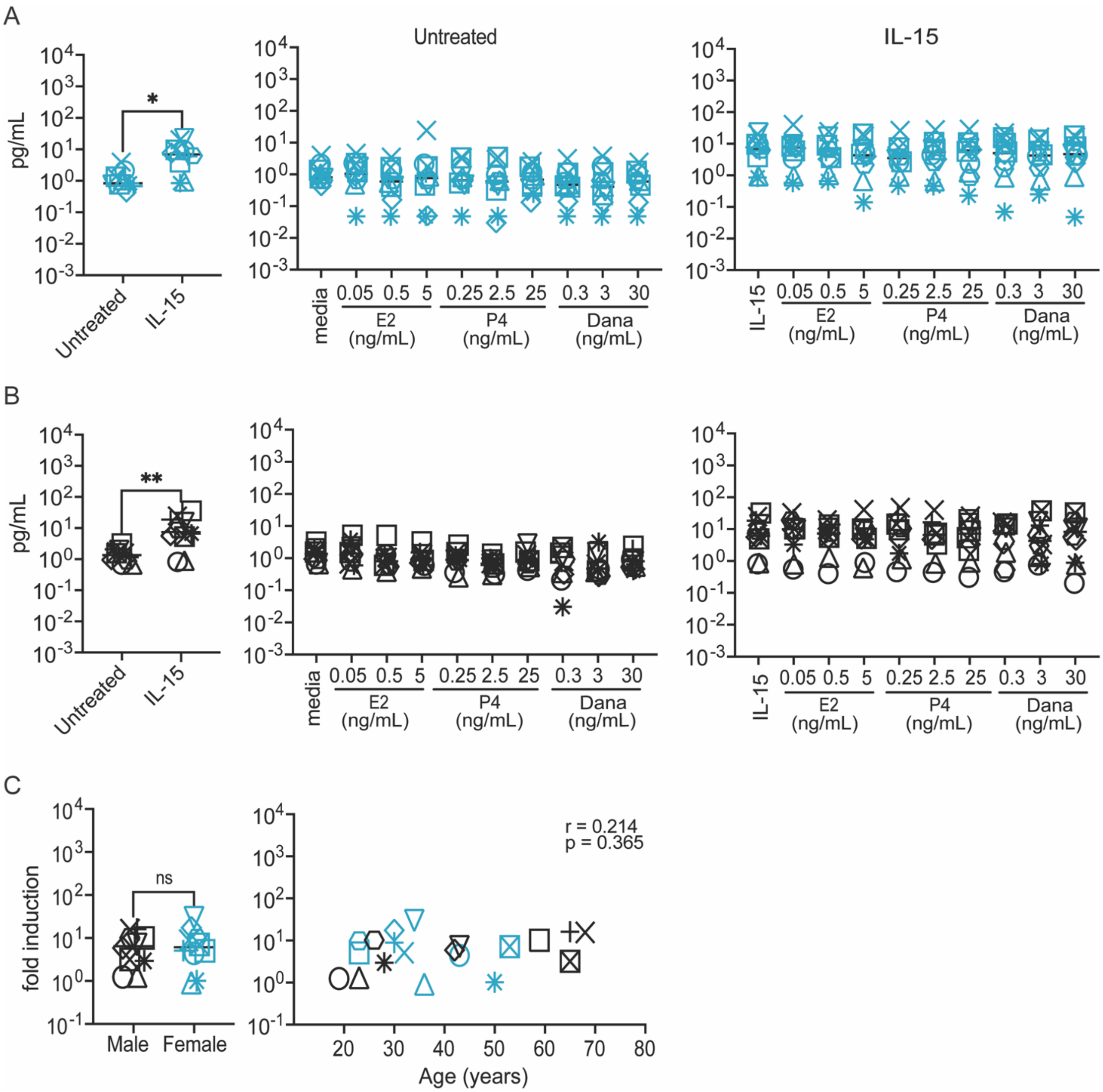

SF12. Analysis of IL-15-mediated IL-4 induction in PBMCs in the presence of sex hormones. IL-4 production before and after stimulation with IL-15 (first panel), hormone treatments of estradiol (E2), progesterone (P4), and danazol (dana) alone (middle panel), or in the presence of IL-15 (right panel). Teal symbols are female donors (A), and black symbols are male donors (B). (C) Fold induction of IL-4 production by biological sex (left) and by age of donor (right). Mann-Whitney test and nonparametric spearman correlation were used to calculate p values (*$p < 0.05$).

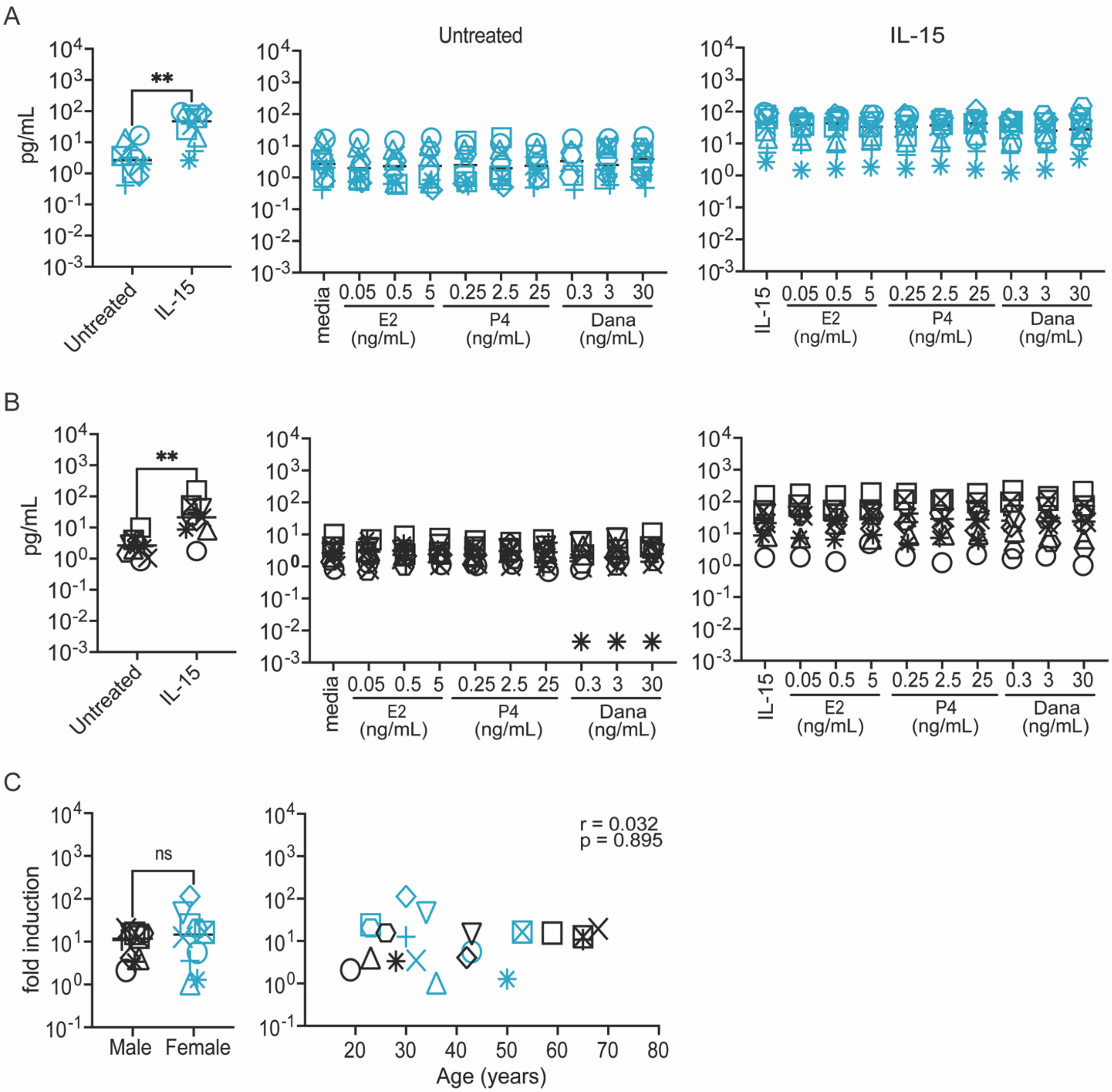

SF13. Analysis of IL-15-mediated IL-10 induction in PBMCs in the presence of sex hormones. IL-10 production before and after stimulation with IL-15 (first panel), hormone treatments of estradiol (E2), progesterone (P4), and danazol (dana) alone (middle panel), or in the presence of IL-15 (right panel). Teal symbols are female donors (A), and black symbols are male donors (B). (C) Fold induction of IL-10 production by biological sex (left) and by age of donor (right). Mann-Whitney test and nonparametric spearman correlation were used to calculate p values (*p < 0.05).

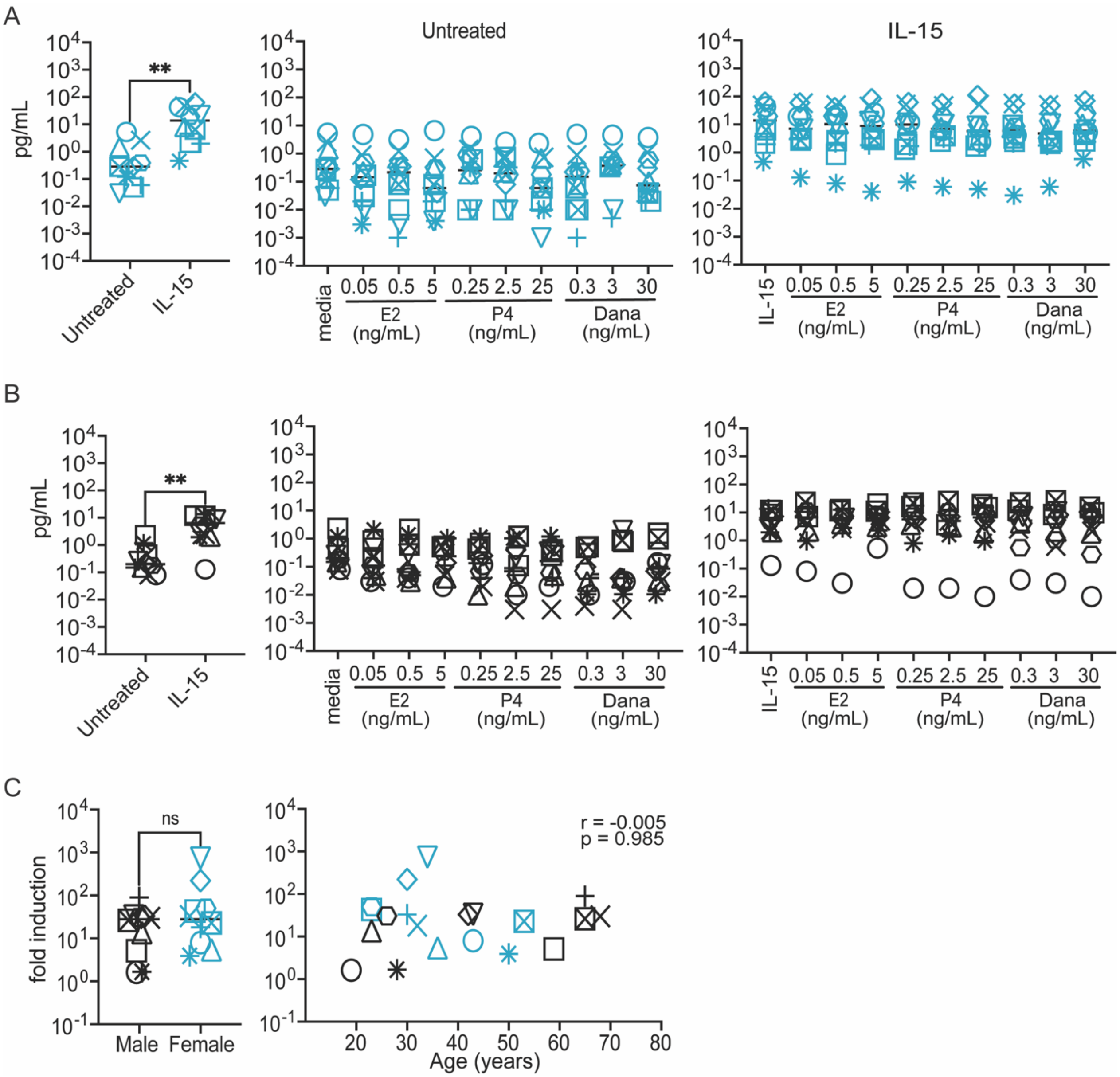

SF14. Analysis of IL-15-mediated IL-5 induction in PBMCs in the presence of sex hormones. IL-5 production before and after stimulation with IL-15 (first panel), hormone treatments of estradiol (E2), progesterone (P4), and danazol (dana) alone (middle panel), or in the presence of IL-15 (right panel). Teal symbols are female donors (A), and black symbols are male donors (B). (C) Fold induction of IL-5 production by biological sex (left) and by age of donor (right). Mann-Whitney test and nonparametric spearman correlation were used to calculate p values (*$p < 0.05$).

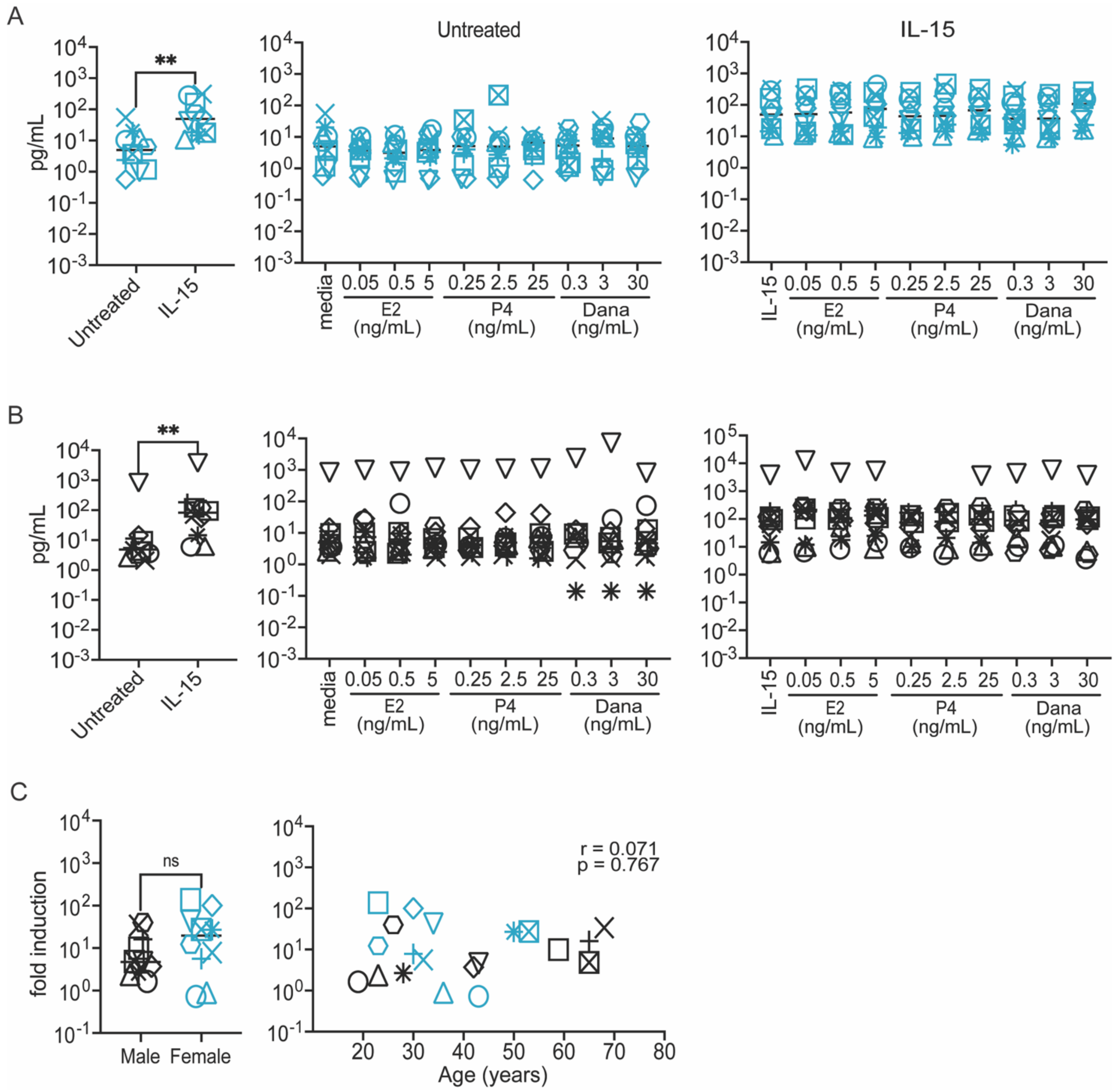

SF15. Analysis of IL-15-mediated IL-6 induction in PBMCs in the presence of sex hormones. IL-6 production before and after stimulation with IL-15 (first panel), hormone treatments of estradiol (E2), progesterone (P4), and danazol (dana) alone (middle panel), or in the presence of IL-15 (right panel). Teal symbols are female donors (A), and black symbols are male donors (B). (C) Fold induction of IL-6 production by biological sex (left) and by age of donor (right). Mann-Whitney test and nonparametric spearman correlation were used to calculate p values (*$p < 0.05$).

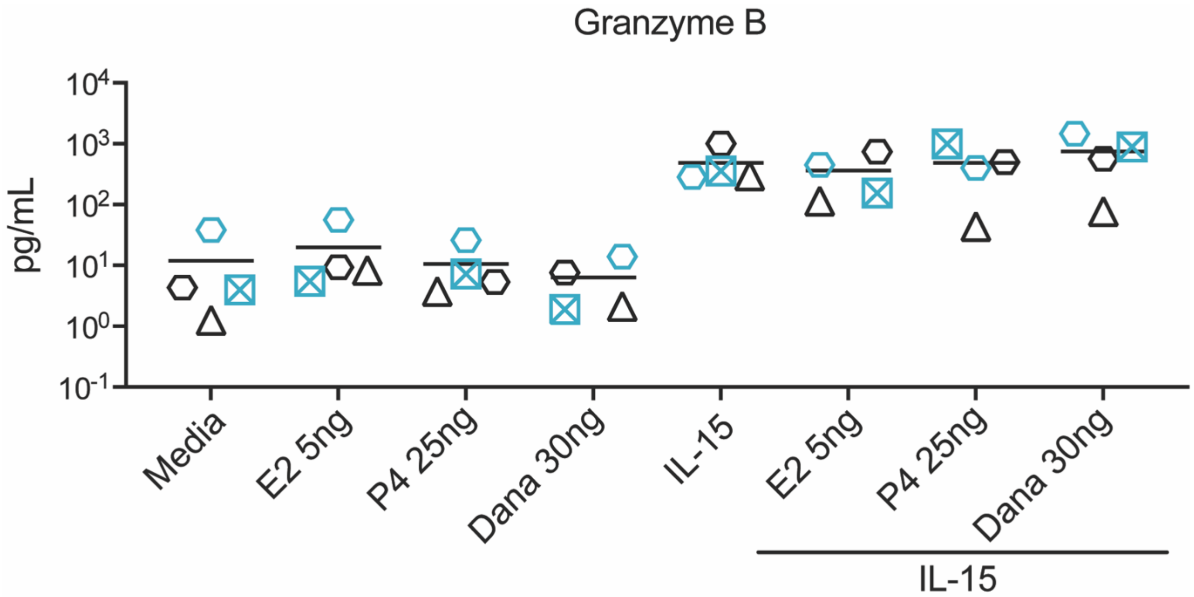

SF16. Analysis of IL-15-mediated granzyme B production in PBMCs in the presence of sex hormones. (A) Granzyme B production with and without IL-15 stimulation in the presence of hormone treatments of estradiol (E2), progesterone (P4), and danazol (dana). Teal symbols are female donors and black symbols are male donors. These donor symbols are the same as in the cytokine panel.

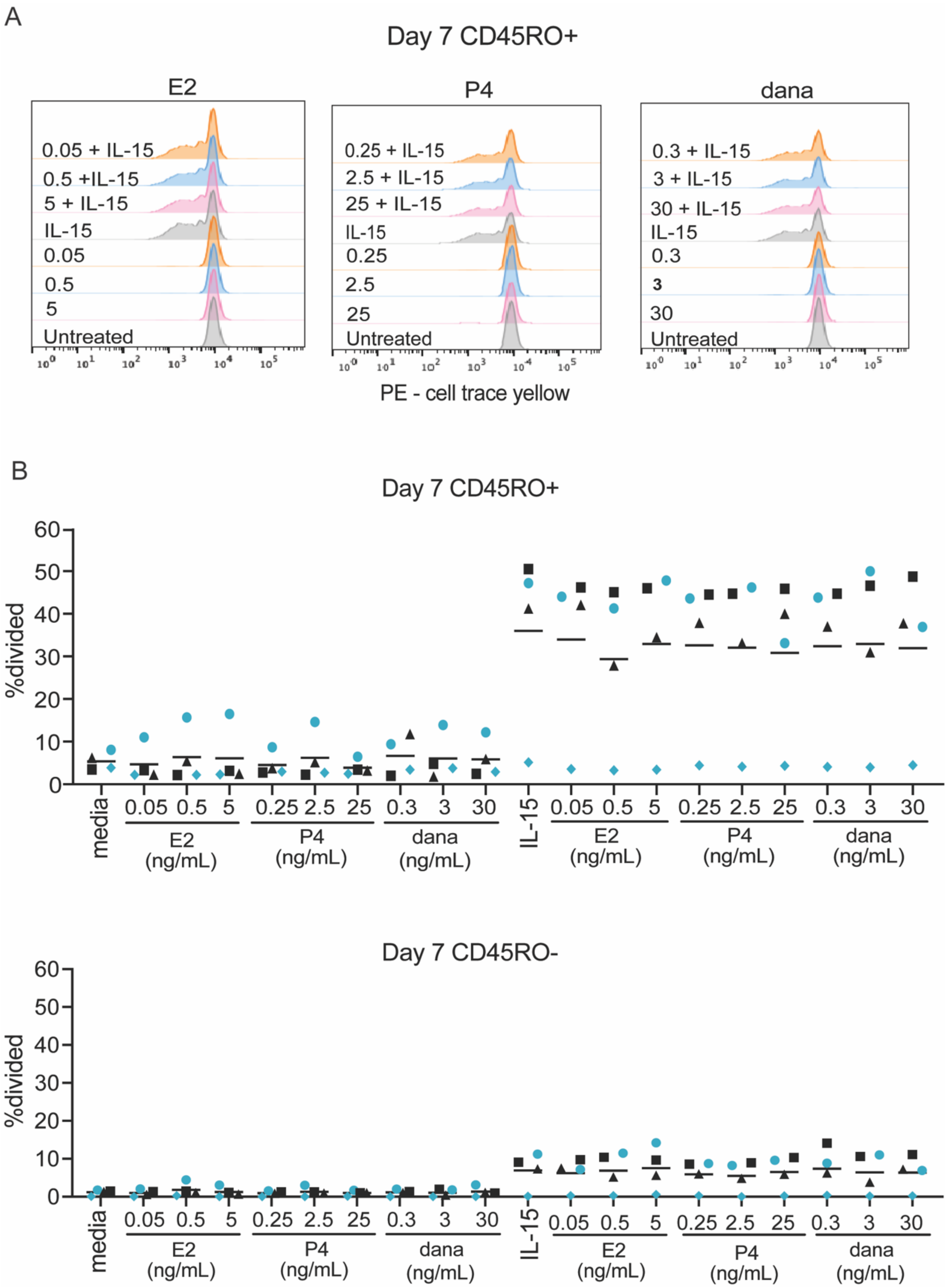

SF17. Analysis of CD4 T cell IL-15-mediated proliferation with E2, P4, and dana. (A) Representative donor showing histograms of proliferation after 7 days in CD4+CD45RO+ cells treated with E2 (left), P4 (middle), or dana (right) with and without IL-15 (100ng/mL). (B) Analysis of proliferation in CD45RO+ (top) and CD45RO- (bottom) after 7 days in CD4+CD45RO+ cells treated with E2 (left), P4 (middle), or dana (right) with and without IL-15 (100ng/mL). Teal symbols are female donors and black symbols are male donors.

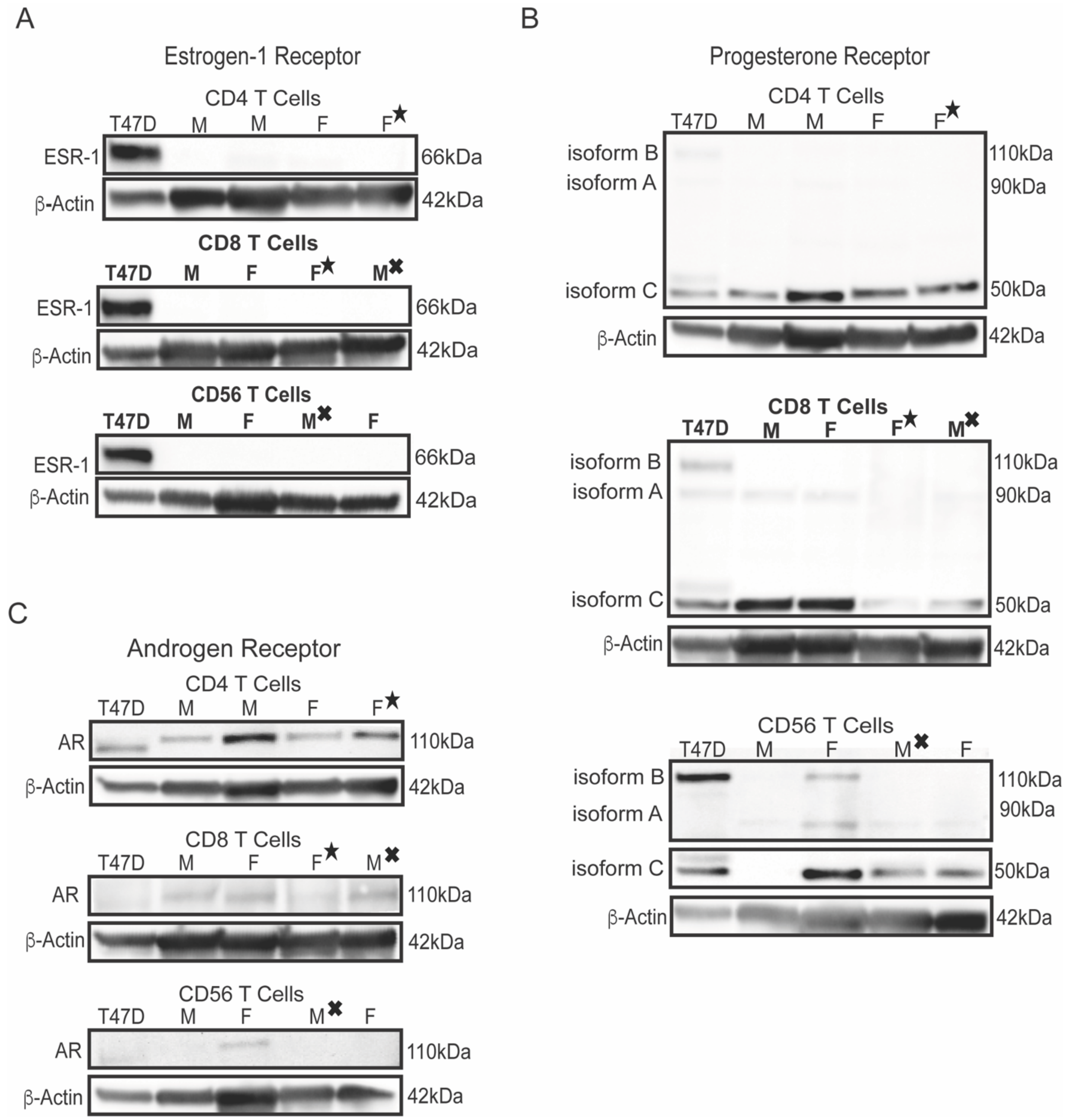

SF18. Western blot analysis for estrogen-1, progesterone, and androgen receptor. Western blot analysis of estrogen receptor-1 (ESR-1 clone D8H8, Cell Signaling Technology, cat#8644S) (A), progesterone receptor (B), and androgen receptor (C), in ESR-1+ breast cancer cell line T47D and CD4 T, CD8 T, and NK cells from two male and two female donors.

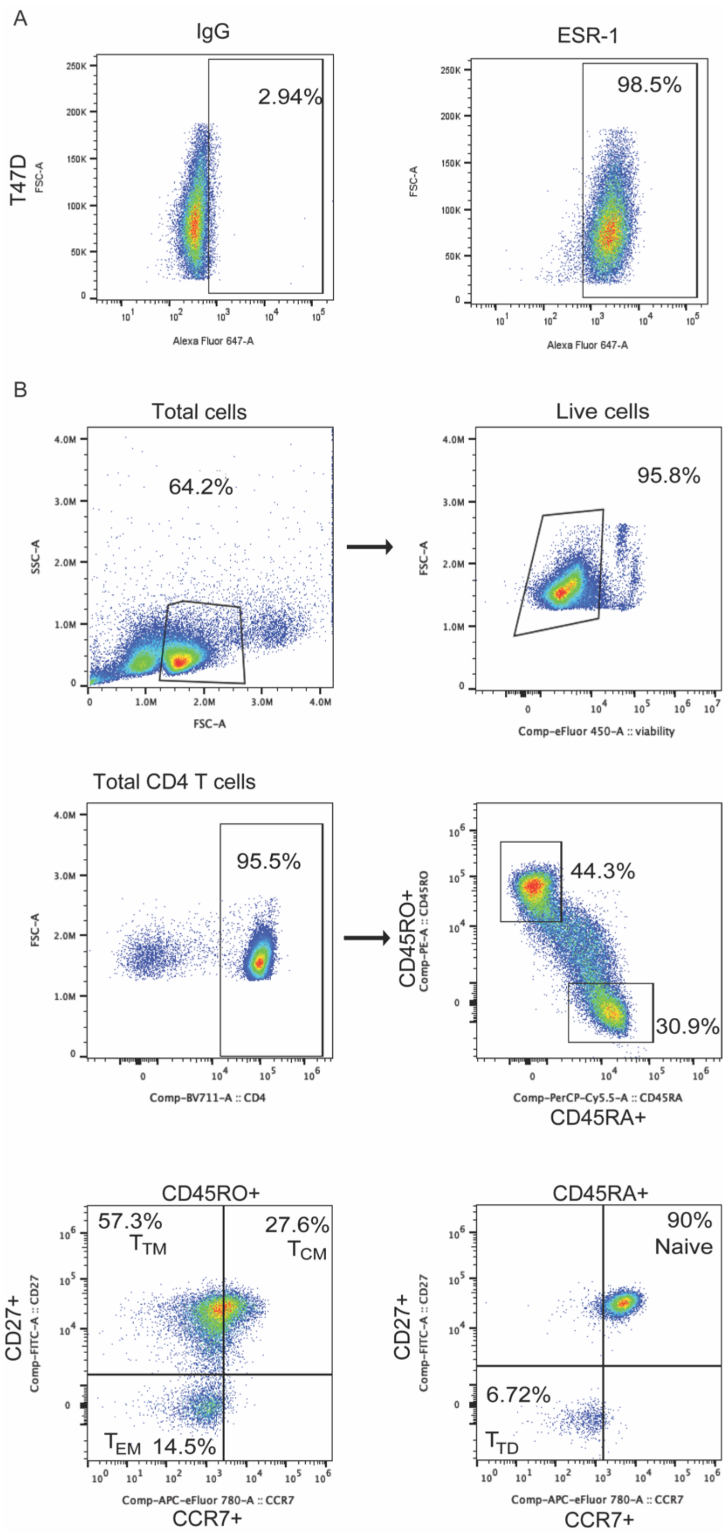

SF19. Gating strategy of T47D breast cancer cell line and represenentive donor showing CD4 T cell subsets.

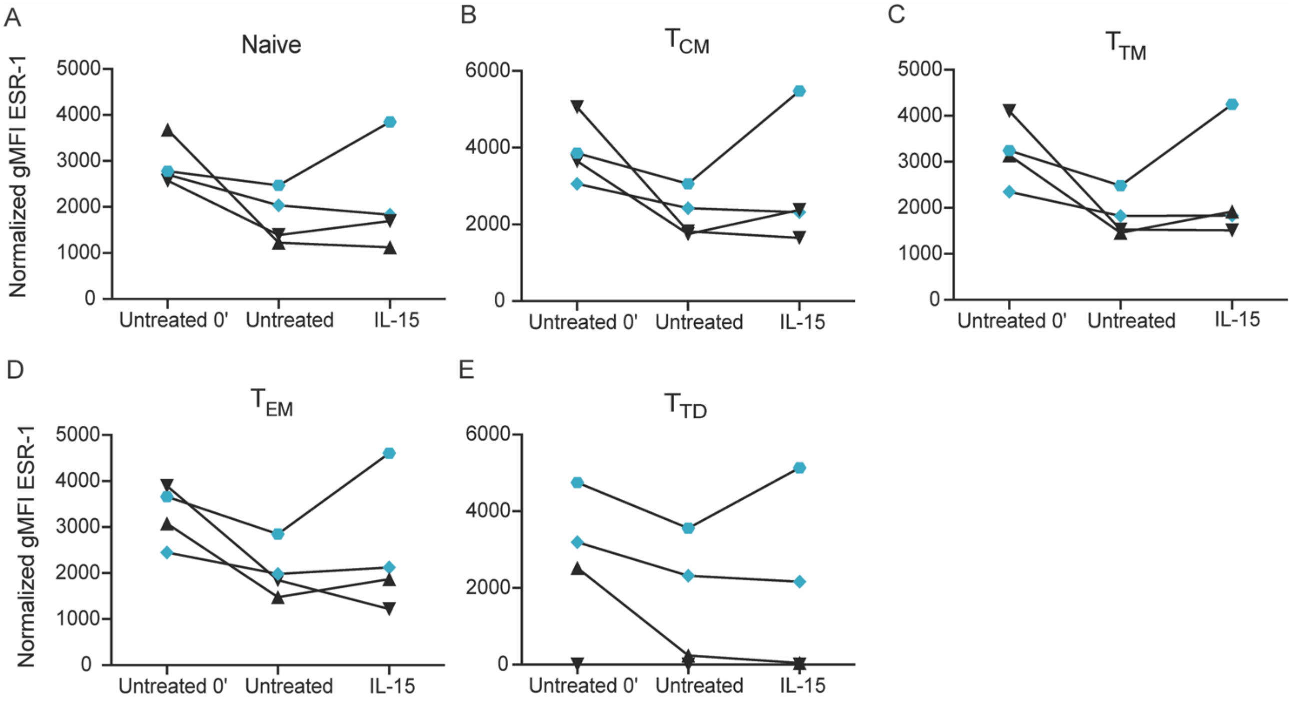

SF20. Analysis of the expression of ESR-1 in CD4 T cell subsets with and without IL-15 treatment. Expression of ESR-1 at 0 hours untreated, overnight untreated, and overnight IL-15 treated in naïve CD4 T cells (A), T_{CM} cells (B), T_{TM} cells (C), T_{EM} cells (D), and T_{TD} cells (E). Teal symbols are female and black symbols are male donors.

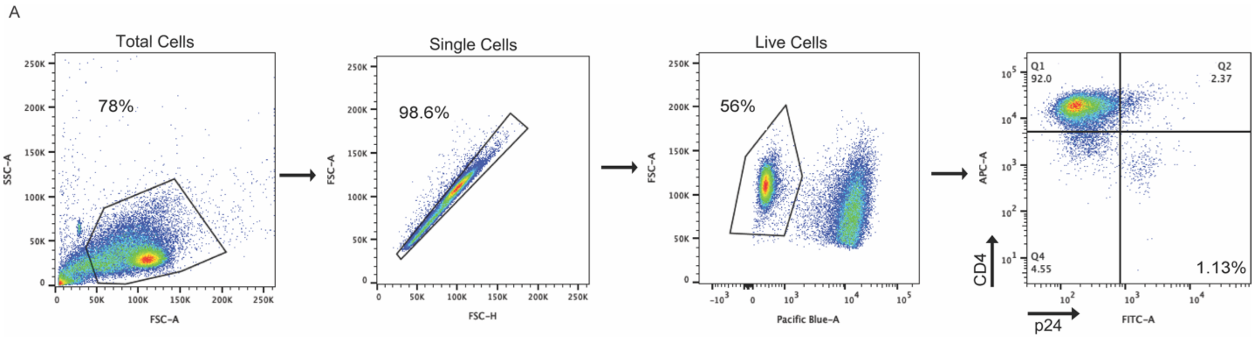

SF21. Gating strategy for the T_{CM} Model of Latency in an untreated representative donor

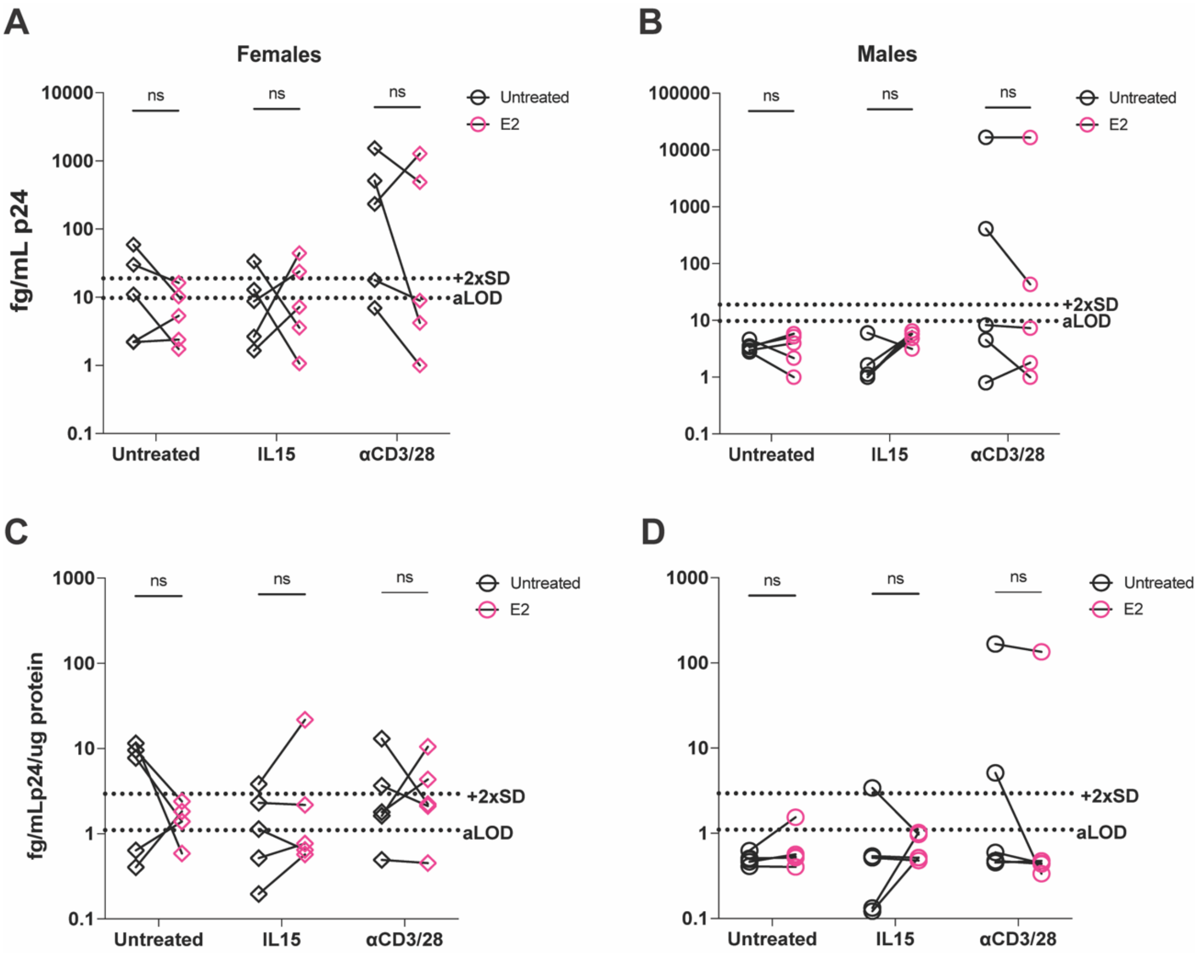

<u>Figure 22.</u> Varying effects of 17β-estradiol on HIV reactivation in ART-suppressed people with HIV. Analysis of figure 5 separated by biological sex showing the viral reactivation in supernatants of females (A) and males (B) and in cell lysates of females (C) and males (D). Pink symbols are with E2 treatment and black symbols are no E2 treatment.

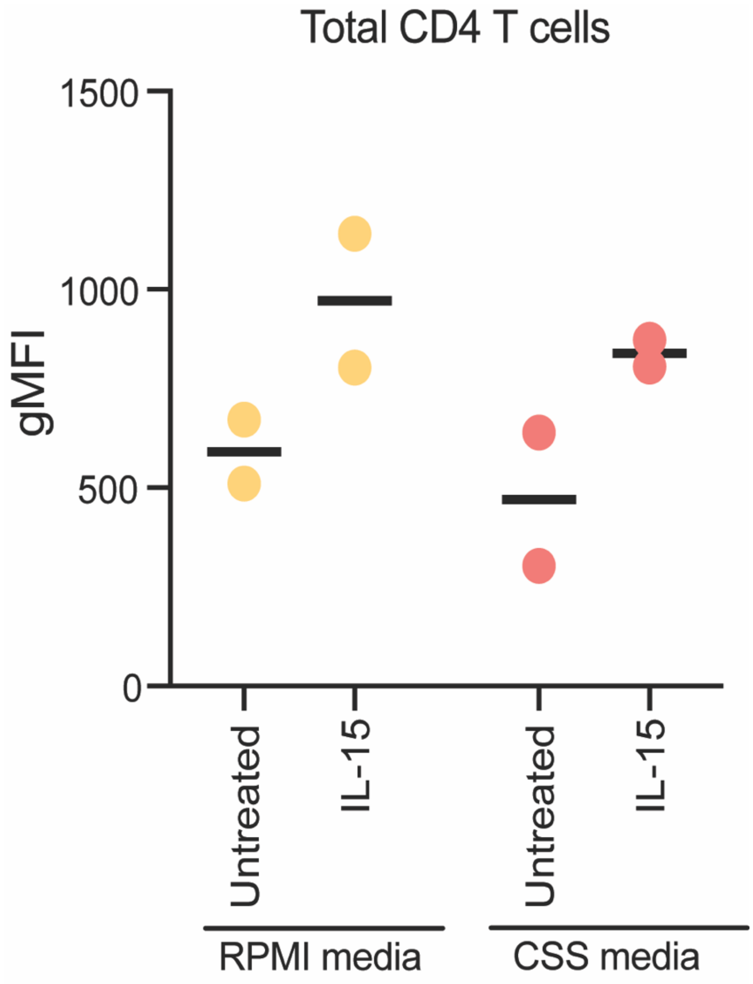

SF23. STAT5 phosphorylation in total CD4 T cells. Flow cytometry measuring pSTAT5 with and without IL-15 stimulation in total CD4 T cells in RMPI media (left) and CSS media (right).

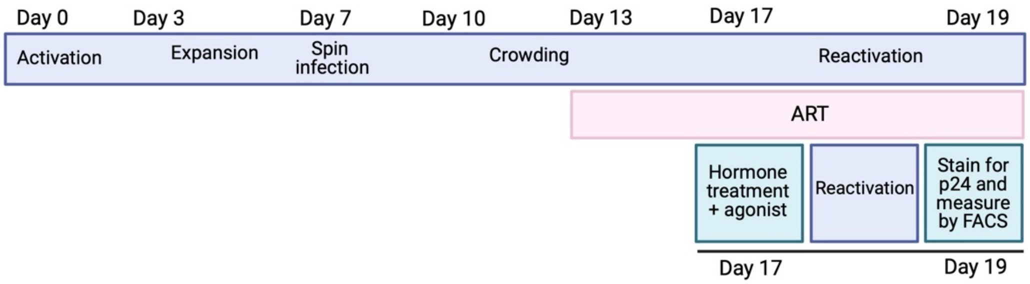

SF24. Timeline of the T_{CM} Model of Latency.
