# Supplementary material for: HIV-1 latency reversal and immune enhancing activity of IL-15 is not influenced by sex hormones
Source: JCI Insight. 2024 Sep 10;9(17):e180609. doi: 10.1172/jci.insight.180609 (PMC11389825; doi:10.1172/jci.insight.180609)

Full unedited blot/gel for Figure 3

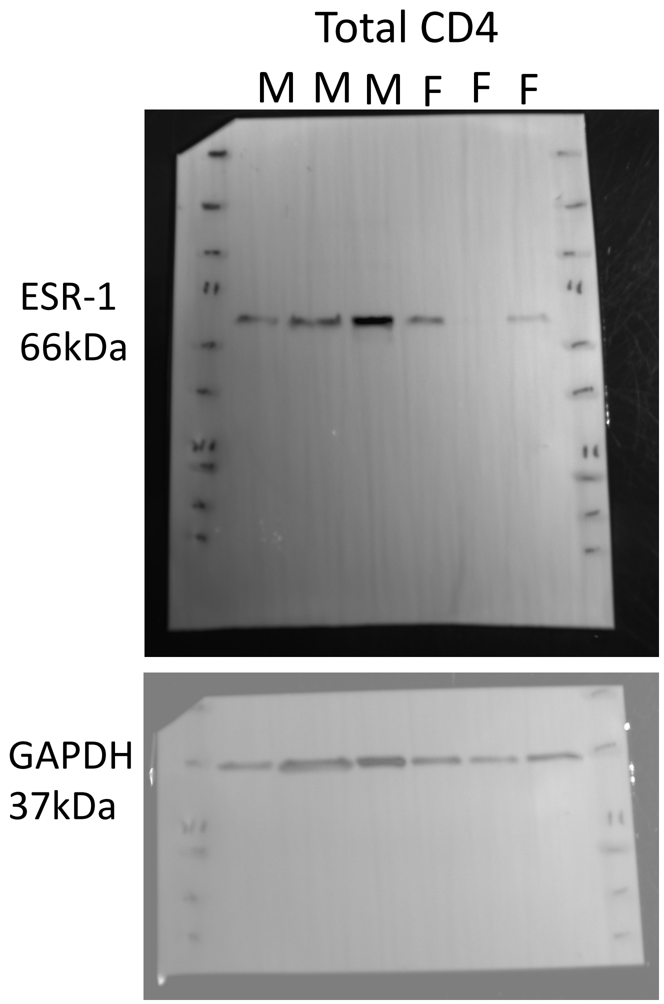

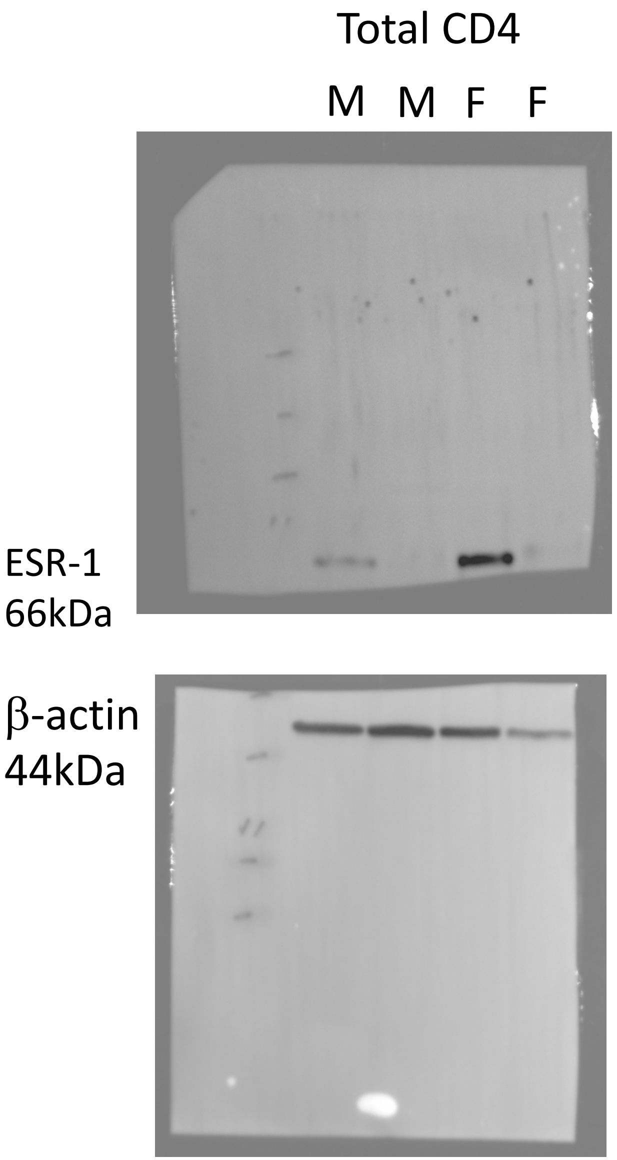

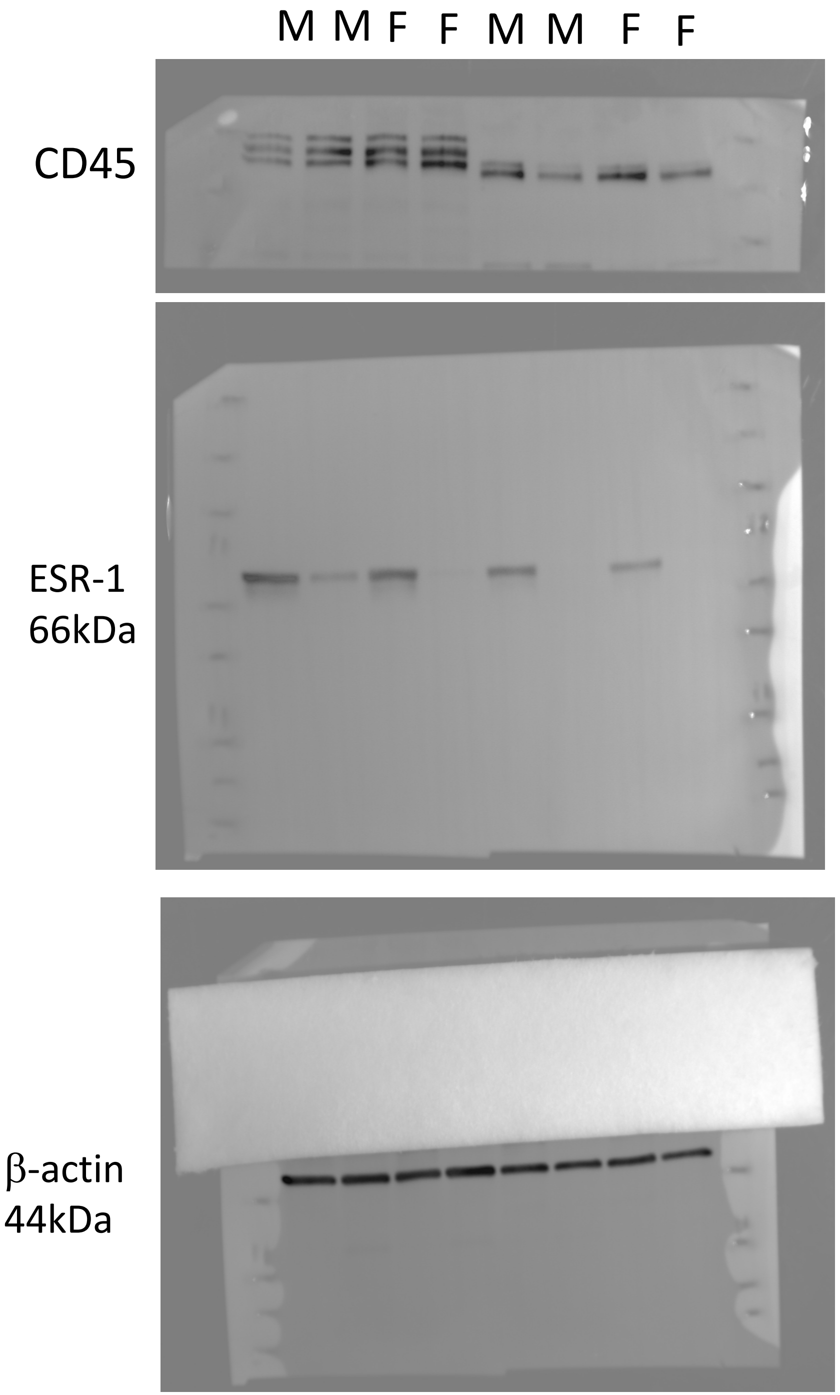

Full unedited blot/gel for Supplemental Figure 2

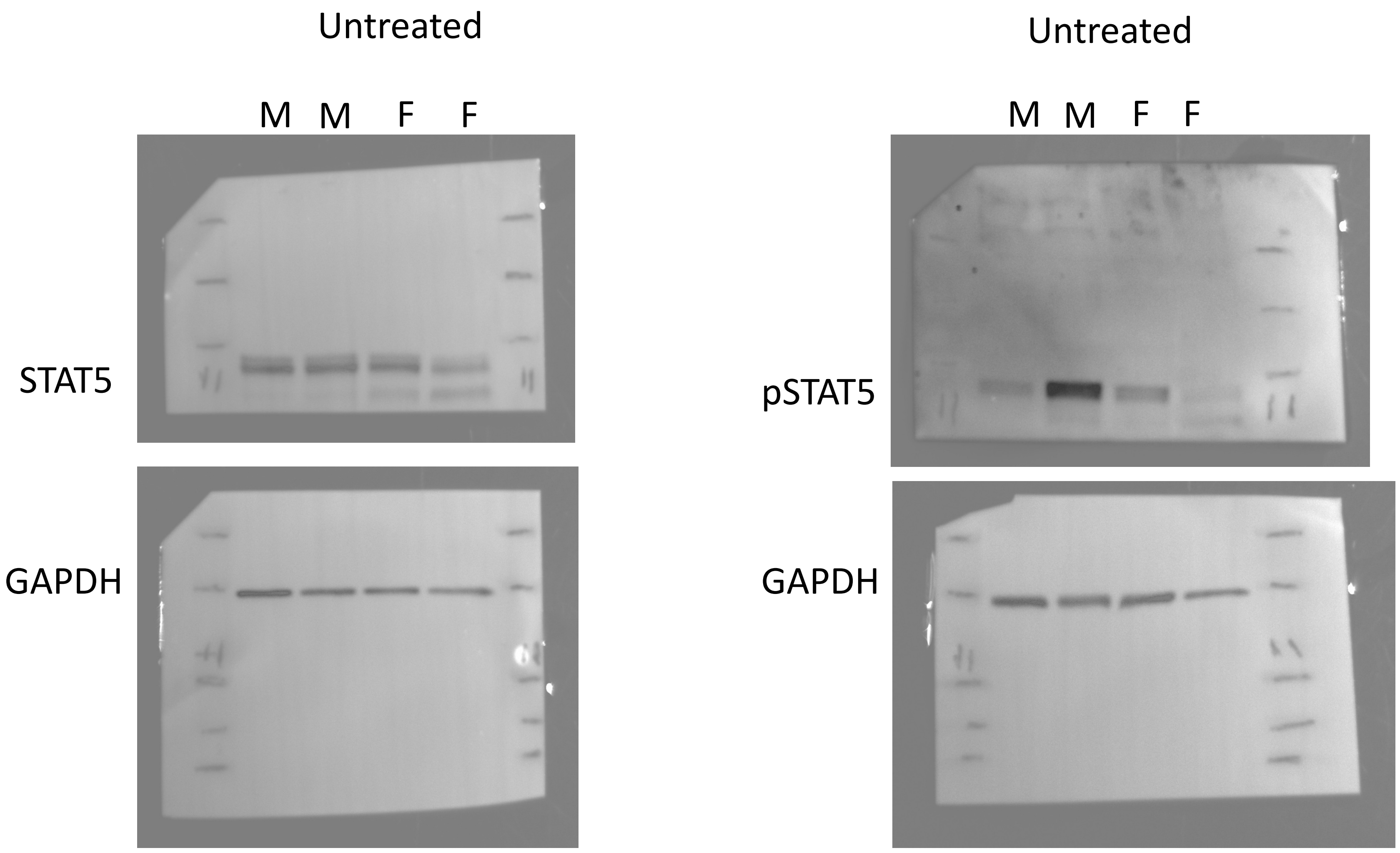

Full unedited blot/gel for Supplemental Figure 2

IL-15 treated

M M F F

STAT5

GAPDH

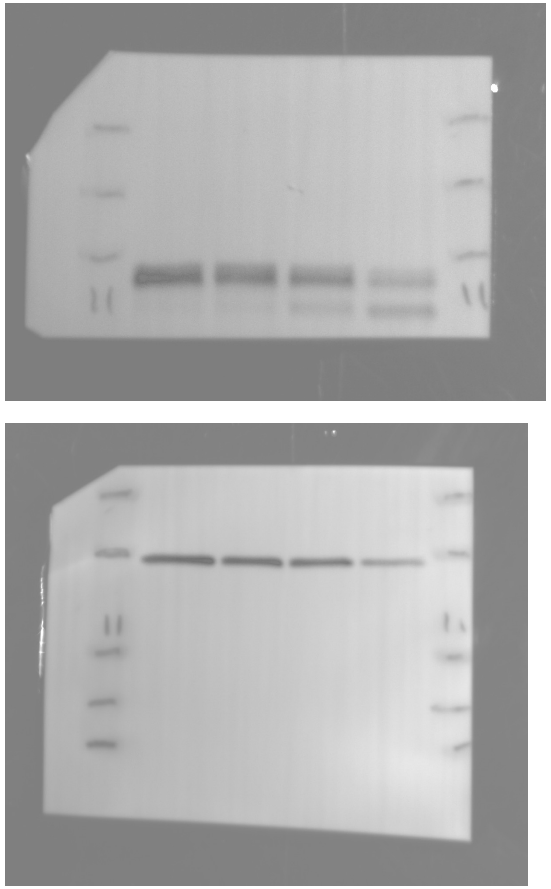

IL-15 treated

M M F F

pSTAT5

GAPDH

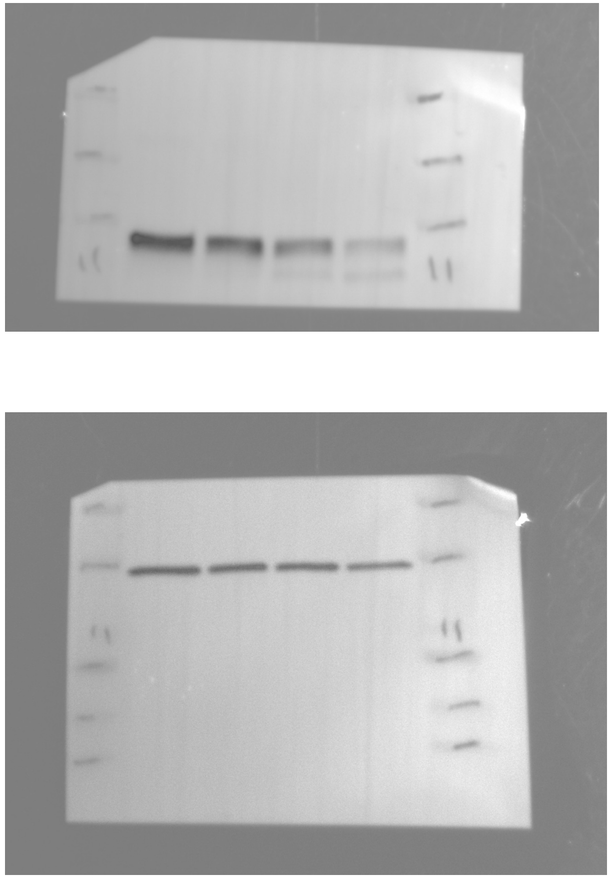

Full unedited blot/gel for Supplemental Figure 14 CD4

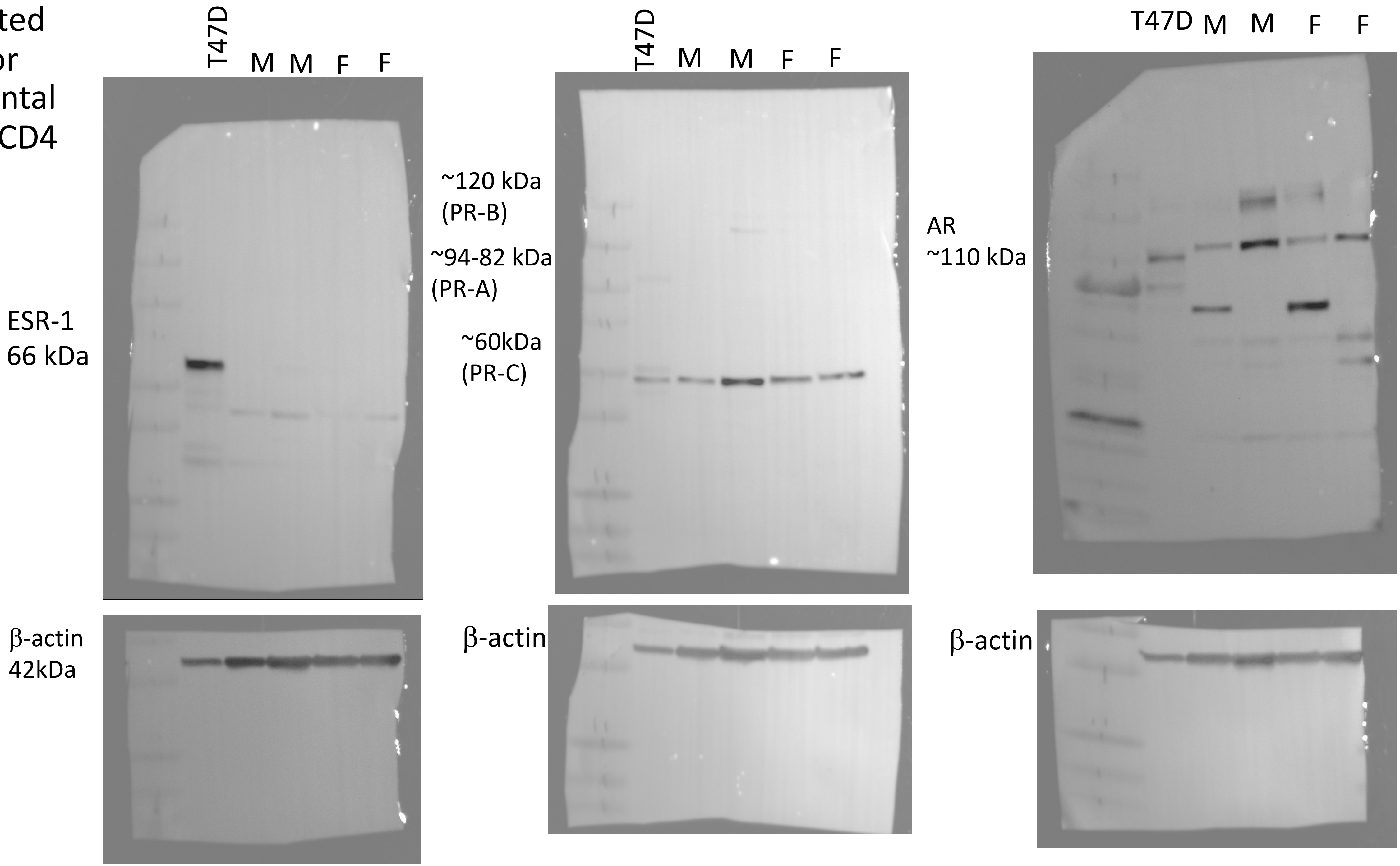

Full unedited blot/gel for
Supplemental Figure 14 CD8

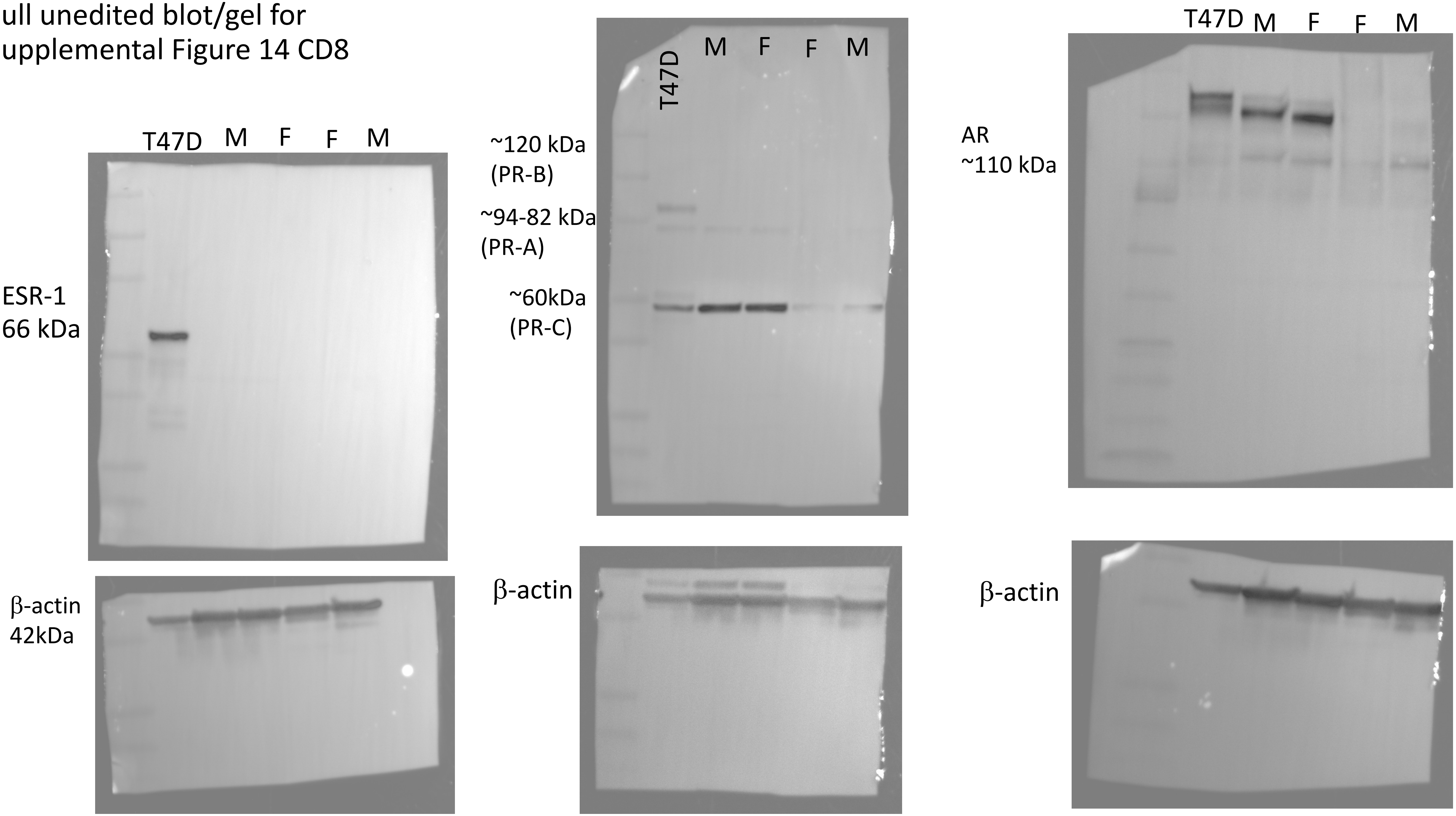

Full unedited blot/gel for Supplemental Figure 14 CD56

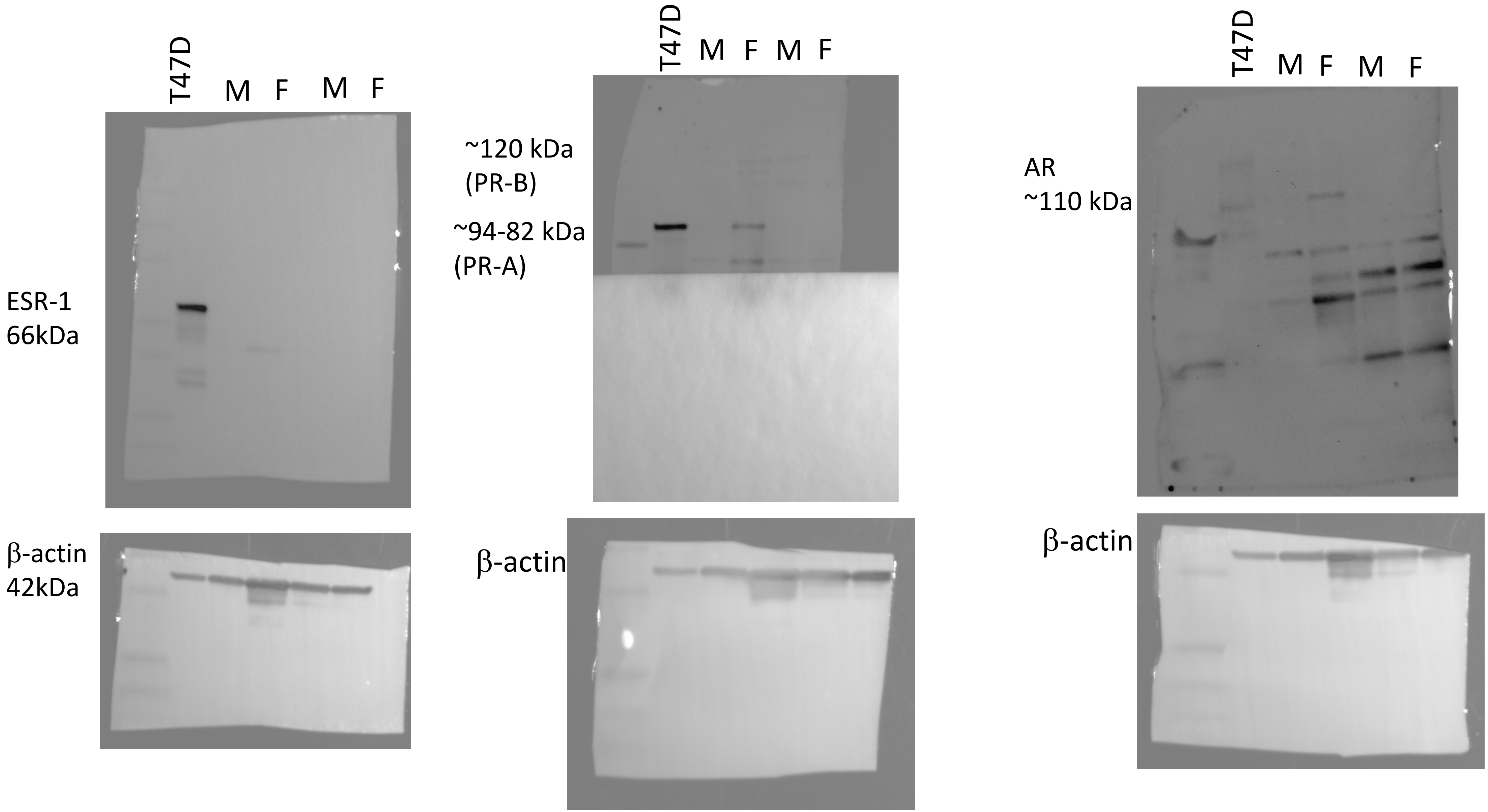

Supplement: Unedited blot and gel images [file jciinsight-9-180609-s007.pdf]
